# Rational Molecular Engineering of Amidonaphthoquinone Cathodes: Precise Hydrogen Bond and Size Control for High‐Performance Lithium Organic Batteries

**DOI:** 10.1002/advs.202505936

**Published:** 2025-05-23

**Authors:** Qianglong Chen, Fangfang Xing, Jia Cai, Xiujuan Wang, Xiaoming He

**Affiliations:** ^1^ Key Laboratory of Applied Surface and Colloid Chemistry (Ministry of Education) School of Chemistry and Chemical Engineering Shaanxi Normal University Xi'an 710119 P. R. China

**Keywords:** amidonaphthoquinone, cathode, hydrogen bond, lithium organic battery, small molecule

## Abstract

Rational design of organic cathode materials with suppressed solubility is crucial yet challenging for achieving high‐capacity and long‐cycling rechargeable batteries. This study presents a facile synthesis strategy for three naphthoquinone derivatives (NQ1‐NQ3) featuring tunable amide functionalities and molecular dimensions, followed by a systematic evaluation of their electrochemical performance in lithium‐organic batteries (LOBs). The strategic incorporation of multiple amide motifs and molecular size expansion in NQ2 and NQ3 effectively enhances intermolecular interactions through hydrogen‐bonding networks and π–π stacking, resulting in remarkable solubility suppression and superior cycling stability. Notably, the NQ3‐based cathode demonstrates an intriguing structural evolution involving progressive particle pulverization during cycling, which facilitates intimate contact with conductive carbon additives and significantly improves electrode conductivity. These synergistic effects enable the best LOB performance of NQ3, such as a high specific capacity (224 mAh g^−1^ at 0.1 A g^−1^), good rate capability (162 mAh g^−1^ at 2 A g^−1^) and cycling stability, outperforming most reported organic cathode materials. This work provides molecular‐level insights into suppressing dissolution through non‐covalent interaction engineering for high performance LOBs.

## Graphical abstract

This work provides a novel molecular design strategy to develop three naphthoquinone‐derived cathodes (NQ1, NQ2, and NQ3) by linking one, two, and three amido‐naphthoquinone redox centers onto a benzene ring in a convenient way. Structure evolution to increased hydrogenbonds and molecular size enables high performance LOBs by joint solubility suppress and morphology reorganization.

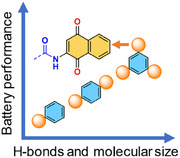

## 1 Introduction

The global transition toward sustainable energy systems has driven relentless growth in demand for eco‐friendly and cost‐effective electrochemical energy storage.^[^ ^1^ ^]^ However, state‐of‐the‐art lithium‐ion batteries (LIBs), which dominate the market, rely heavily on the based on transition‐metals based electrode chemistry. These materials face intrinsic limitations, such as scarce resources, elevated production costs, and environmentally detrimental extraction/disposal processes. Furthermore, conventional inorganic electrodes are approaching their theoretical capacity ceilings, posing a critical bottleneck for next‐generation high‐performance rechargeable battery technologies. This situation has catalyzed significant research efforts in recent years to develop sustainable alternatives by using less toxic, cheap, and environmentally benign redox‐active organic materials.^[^ ^2^, ^3^, ^4^, ^5^ ^]^ Through strategic molecular engineering, diverse redox‐active moieties, such as quinone,^[^ ^6^, ^7^, ^8^, ^9^, ^10^, ^11^, ^12^, ^13^ ^]^ viologen,^[^ ^14^, ^15^, ^16^, ^17^ ^]^ carbonylpyridinium,^[^ ^18^, ^19^, ^20^, ^21^ ^]^ azobenzene,^[^ ^22^, ^23^, ^24^, ^25^ ^]^ phenothiazine^[^ ^26^, ^27^, ^28^, ^29^ ^]^ and phenazine,^[^ ^30^, ^31^, ^32^, ^33^, ^34^, ^35^ ^]^ have been identified as building blocks for construction of organic electrodes.

Despite these advancements, organic electrode materials face a formidable obstacle: undesirable dissolution in liquid electrolytes. While polymerization has emerged as a widely adopted strategy to suppress solubility issues, conventional synthetic approaches typically yield polydisperse polymer mixtures, and often bring up batch‐to‐batch variations that can hamper reproducibility. Alternative physical confinement strategies, such as immobilizing organic materials in mesoporous carbon matrices,^[^ ^36^ ^]^ introduce practical limitations through elevated production costs and compromised energy density. These limitations collectively underscore a critical need for innovative molecular design that capitalize on the unique advantages of precisely engineered molecular architectures, thereby enabling simultaneous achievement of reproducibility and systematic optimization of electrochemical properties.

To effectively reduce the solubility of organic compounds, strategic enhancement of intermolecular interactions serves as a fundamental principle. Expanding conjugated structures represents a well‐established method to intensify π–π stacking interactions, which have been widely demonstrated in the polymer design to decrease the solubility.^[^ ^37^ ^]^ Concurrently, hydrogen bonding, a ubiquitous non‐covalent interaction in biological systems with bond dissociation energies ranging from 5 to 120 kJ mol⁻¹, offers complementary opportunities for solubility control.^[^ ^38^, ^39^ ^]^ While individual hydrogen bonds exhibit relatively weak interactions, the deliberate construction of multiple hydrogen bonding networks can significantly amplify molecular cohesion. This synergistic approach becomes particularly potent when combined with π–π stacking and other secondary interactions (e.g., van der Waals forces), creating a multidimensional interaction matrix that substantially suppresses compound dissolution. Despite their potential for designing high‐performance organic electrodes, hydrogen‐bonding strategies remain markedly underexplored, with few examples reported to date.^[^ ^40^, ^41^, ^42^, ^43^ ^]^

In this study, we developed three novel naphthoquinone derivatives (NQ1‐NQ3) through rational molecular engineering for LOB applications. The design strategy features deliberate integration of multiple amide moieties as hydrogen‐bonding motifs coupled with controlled molecular expansion in NQ2 and NQ3, which synergistically strengthens intermolecular interactions via 3D hydrogen‐bonding networks and enhanced π–π stacking. This structural optimization effectively suppresses solubility issues while achieving exceptional cycling stability. Particularly noteworthy is the NQ3‐based cathode, which undergoes electro‐reorganization during electrochemical cycling. The progressive particle refinement mechanism promotes intimate interfacial contact with conductive carbon matrices, resulting in substantially improved charge transfer kinetics. Among the designed molecules, NQ3 exhibits superior electrochemical performance, delivering the highest specific capacity, enhanced rate capability, and optimal cycling durability. This work establishes an innovative molecular design paradigm for developing high‐performance naphthoquinone‐based cathode materials in advanced LOB systems.

## 2 Results and Discussion

### 2.1 Design, Synthesis and Characterization

**Figure 1:**
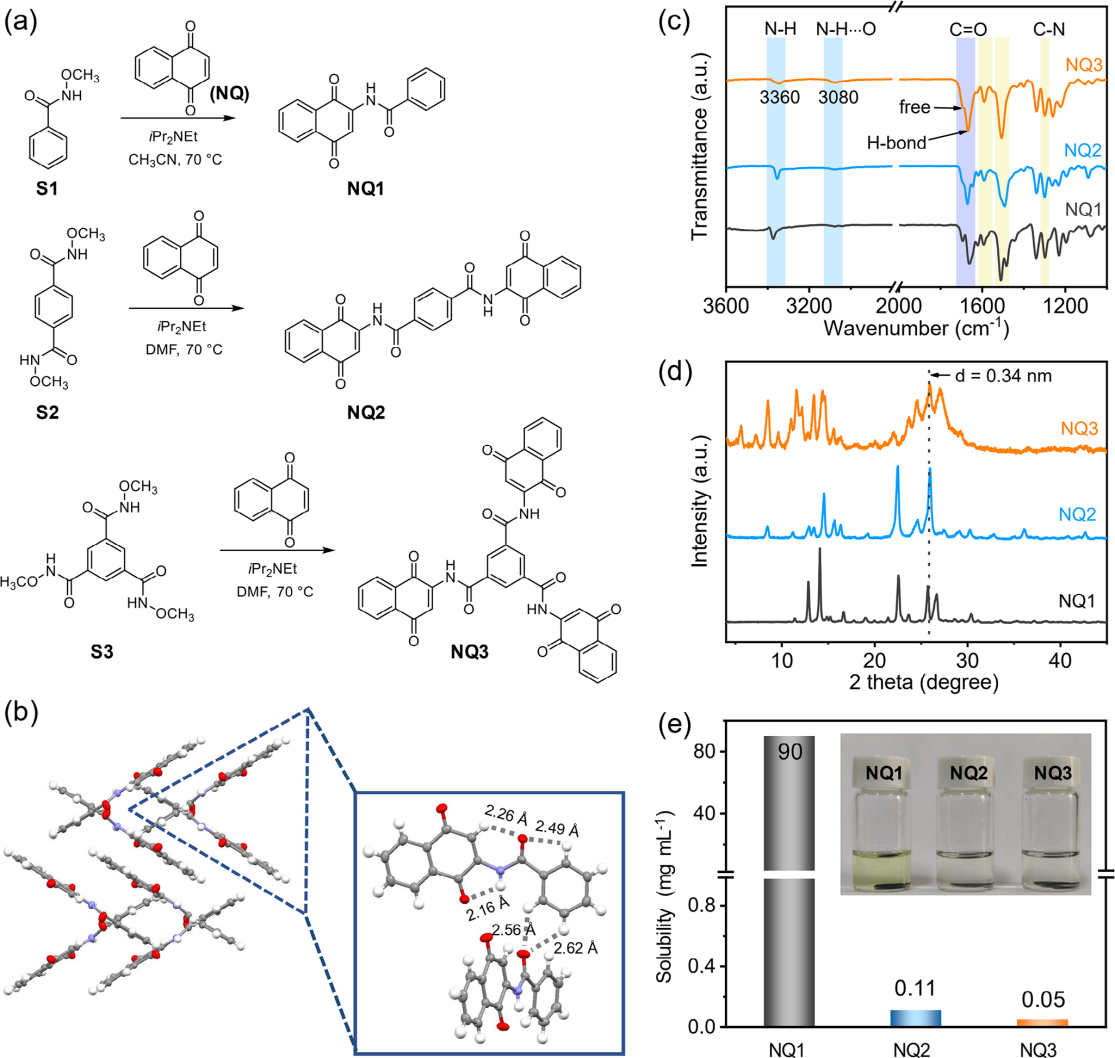
a) Synthesis of NQ1‐NQ3. b) X‐ray crystal structure of NQ1 (left) and the detail of hydrogen bond in the solid state (right). c) FT‐IR spectra and d) XRD patterns of NQ1‐NQ3. e) Solubility of NQ1‐NQ3 in DOL/DME (1:1, v/v) electrolytes. Inset shows the digital photographs of the corresponding DOL/DME (1:1, v/v) electrolytes with one piece of NQ1, NQ2, and NQ3 electrodes for 3 days.

The synthetic procedure toward NQ1‐NQ3 is provided in **Figure** **1**a. Three molecules were prepared in high yields by direct reaction of 1,4‐naphthoquinone (NQ) with O‐methylhydroxamic acids in the presence of *i*Pr_2_NEt as base, according to a modified procedure developed by Zhang et al.^[^ ^44^ ^]^ We used dimethylformamide (DMF) as the solvent, in which all the starting materials can be well dissolved. NQ1 showed good solubility in DMF and can be separated in 85% yield, suggesting the high efficiency of such reaction. In contrast, NQ2 and NQ3 precipitated out during the reaction, implying its poor solubility. Such limited solubility allows their easy purification by hot filtration and washing with DMF and acetone. The efficient direct amidation of NQ, simple purification procedure, together with cheap starting materials, allow us prepare the target molecules in grams scale cost‐effectively.

The structures and purities of target molecules are first confirmed by ^1^H and ^13^C nuclear magnetic resonance (NMR) spectroscopy (Figures S1–S6, Supporting Information). Unlike NQ1 that can dissolve in most common deuterated solvents, the ultralow solubility of NQ2 and NQ3 causes great challenge for the NMR measurement. Interestingly, it was found that addition of strong acid CF_3_COOD can significantly enhance the solubility of NQ2 and NQ3. This could be rationalized by that CF_3_COOD can efficiently break the intermolecular hydrogen bond and encourage the solubility, even in CDCl_3_. Three molecules show well‐resolved ^1^H NMR spectra, clearly demonstrating the good purity. In addition, high resolution mass spectroscopy (HRMS) test further verify the molecular weight of NQ1 and NQ2 (Figures S7 and S8, Supporting Information). Probably as a result of the extremely low solubility, the molecular mass corresponding to NQ3 was not detected.

The structure of NQ1 was unambiguously confirmed by single‐crystal X‐ray crystallography (Figure 1b). The crystal data and structural refinement parameters are summarized in Table S1 (Supporting Information). Typically, three kinds of hydrogen bonds exist in the NQ1 structure, including intramolecular N─H···O (2.16 Å) and C─H···O (2.49 and 2.26 Å), and intermolecular C─H···O (2.56 and 2.62 Å). The intramolecular hydrogen bonds lock the rotation of the molecule, leading to a planar structure. Intermolecular π–π stacking with d space of 3.4 Å is also observed. Despite all attempts to grow single crystals of NQ2 and NQ3 were met with failure, it is reasonable to believe multiple hydrogen bonds and π–π stacking also exist.

The structure of three molecules was also characterized by Fourier transform infrared spectroscopy (FT‐IR) and Powder X‐ray diffraction (XRD). In the FT‐IR spectra (Figure 1c), the two peaks located at ≈ 3360 and 3080 cm^−1^ are assigned to stretching vibrations of N─H and N─H···O, supporting the existence of hydrogen bonding interaction in NQ1‐NQ3. Consistent information can be garnered from the splitting of C═O vibration peak at ≈ 1670 cm^−1^. A clear stretching vibration peak around 1230 cm^−1^ refer to the signal of C‐N bonds, proving the successful formation of the amide groups in in NQ1‐3. The results are good proofs of the successful synthesis of target products, and corroborate the existence of hydrogen bonds. XRD analyses confirm the crystallinity of three molecules (Figure 1d). Notably, XRD patterns of three molecules reveal signals at 2θ of ≈ 25.8° with a d‐spacing of around 0.34 nm, indicating the presence of π–π interactions in the solid state. Compared to NQ1 and NQ2, NQ3 exhibits more diffraction peaks, probably due to its complicated molecular stacking as a result of the enlarged molecular size and multiple hydrogen bonds. NQ1‐3 show excellent thermal stability, with their decomposition temperatures (*T*_d_) determined to be above 233, 332, and 340 °C under N_2_, respectively (Figure S9, Supporting Information). Obviously, the thermal stability has been significantly improved as the molecular size and numbers of hydrogen bond increase.

As an important factor influencing the cycling performance, the solubility of three molecules in electrolyte were investigated. After soaking the electrodes in dioxolane/dimethoxyethane (DOL/DME, 1:1, v/v) solution (our LOB electrolyte) for 3 days, the solution of NQ1 displayed a light‐yellow color, while the solutions of NQ2 and NQ3 electrodes remained colorless (Figure 1e). Furthermore, the solubility of three molecules in DOL/DME electrolyte was accurately measured by UV spectrophotometry (Figure S10, Supporting Information). As summarized in Figure 1e, NQ1 has extremely high solubility of 90 mg mL^−1^, while the solubilities of NQ2 and NQ3 are only 0.11, and 0.05 mg mL^−1^, respectively. The extremely diminished solubility of NQ2 and NQ3 is a direct consequence of enlarged molecular size with enhanced intermolecular π–π stacking and hydrogen bonding interactions, validating our design concept.

### 2.2 Electrochemical Properties and Calculation

**Figure 2:**
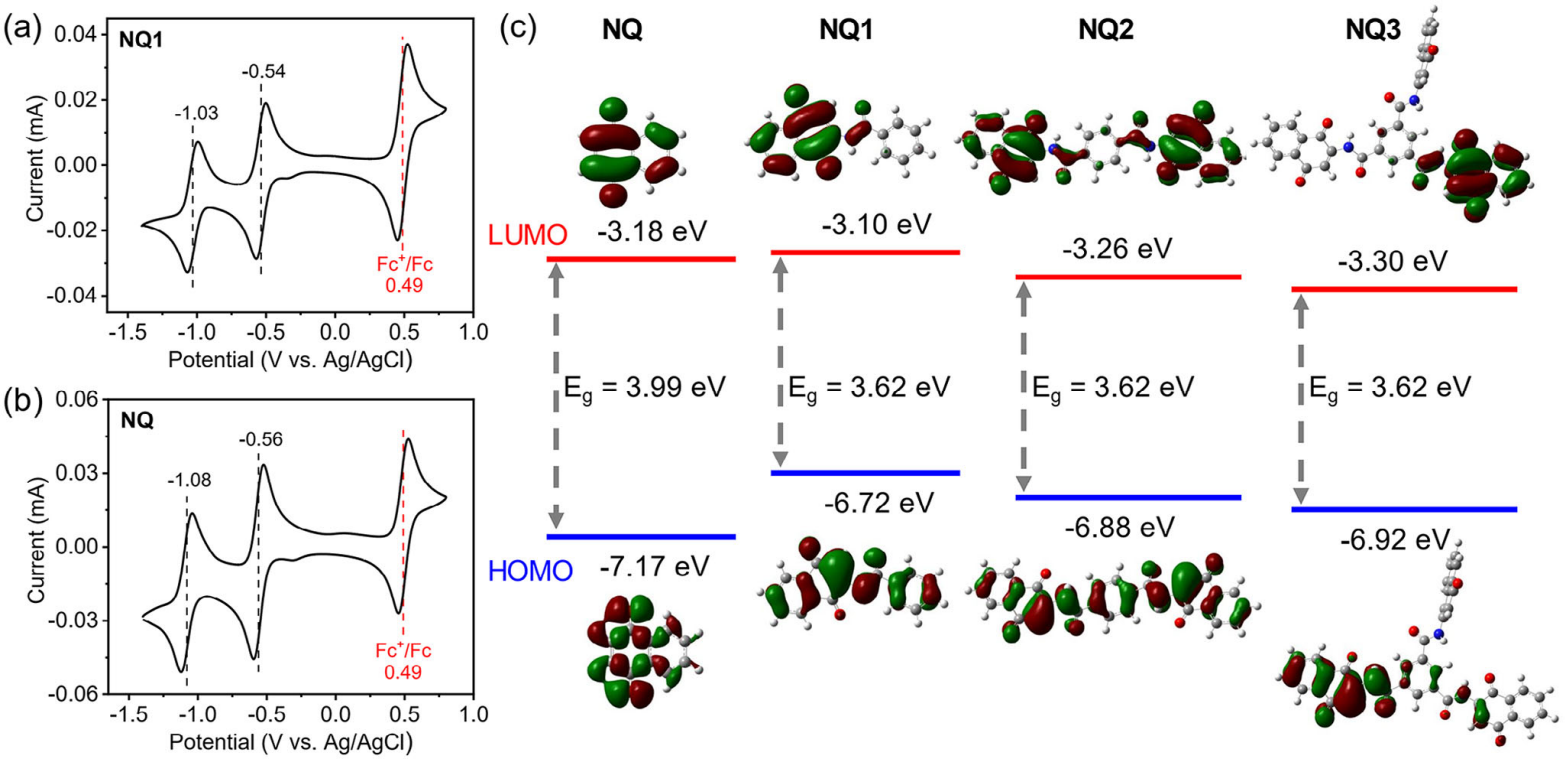
CV curves of a) NQ1 and b) NQ in CH_3_CN solution (*c* = 1 mm) with 0.1 m TBAPF_6_ as the electrolyte. c) Calculated HOMO, LUMO energy levels, and energy gaps of NQ, NQ1, NQ2, and NQ3.

The electrochemical properties of NQ1 and unsubstituted NQ were initially evaluated using cyclic voltammetry (CV) in acetonitrile with 0.1 M tetrabutylammonium hexafluorophosphate (TBAPF_6_) as a supporting electrolyte. As shown in **Figures** **2**a,b and S11 (Supporting Information), NQ1 demonstrates two reversible reductions at −0.54 and −1.03 V (vs Ag/AgCl), and the profile is almost identical to that of NQ. This result confirms that modification of NQ with amide group would not alter the two redox property. Due to the insolubility of NQ2 and NQ3 in common solvents, we were unable to measure their CV data in solution.

DFT calculations were performed to study the electronic properties of three NQs. Figure 2c depicts frontier molecular orbitals and their energy levels. The lowest unoccupied molecular orbital (LUMO) of all the NQs are located on the naphthoquinone moieties, verifying its role on accepting electrons. Comapred to NQ, NQ1 has a slightly higher LUMO energy, implying the weak electron‐donating propety of amide group. In addition, the LUMO energy levels underwent gradual and slight lowering down from NQ1 to NQ3, as a result of the spatial effect from the increased electron‐withdrawing NQ units. According to molecular orbital theory,^[^ ^28^, ^45^ ^]^ a lower LUMO energy indicates a larger electron affinity and higher oxidizing ability, which would result in higher the discharge voltage in battery. Due to the non‐conjugated amide linker, NQ1‐NQ3 show the same energy gaps (*E*_g_) of 3.62 eV. Nevertheless, the *E*_g_ of three amide functionalized molecules is smaller than unsubstituted NQ, as a result of increased conjugation. A lower *E*_g_ is expected to endow better charge transfer ability and accordingly promote fast redox reaction. Moreover, the calculated electrostatic potential (ESP) was used to identify the redox‐active sites of NQ1‐NQ3. As shown in Figure S12 (Supporting Information), multiple negative regions are mainly located around C═O groups around O atoms, indicating their higher electronegativity compared to the other regions. These high electronegative regions easily capture more cations and contribute to high theoretical capacity.

### 2.3 Battery Performance

To investigate the LOB performance, coin cells were assembled by pairing the organic cathodes with lithium metal anodes, and filled with 1.0 m lithium bistrifluoromethanesulfonimide (LiTFSI) in DOL/DME (1:1, v/v) as the electrolyte. The tested cathodes consisting of 60 wt.% designed molecules as active materials, 30 wt.% ketjin black (ECP‐600J) as conductive additive and 10 wt.% poly(vinylidene fluoride) (PVDF) as binder. Taking into consideration the well‐known two‐electron redox property per each naphthoquinone unit, the theoretical capacities (*C*_thero_) for NQ1, NQ2 and NQ3 are calculated to be 194, 225, and 238 mAh g^−1^, respectively. The gradual increased C_thero_ values are related to the higher density of redox unit when grafting more NQ units into one benzene core.

**Figure 3:**
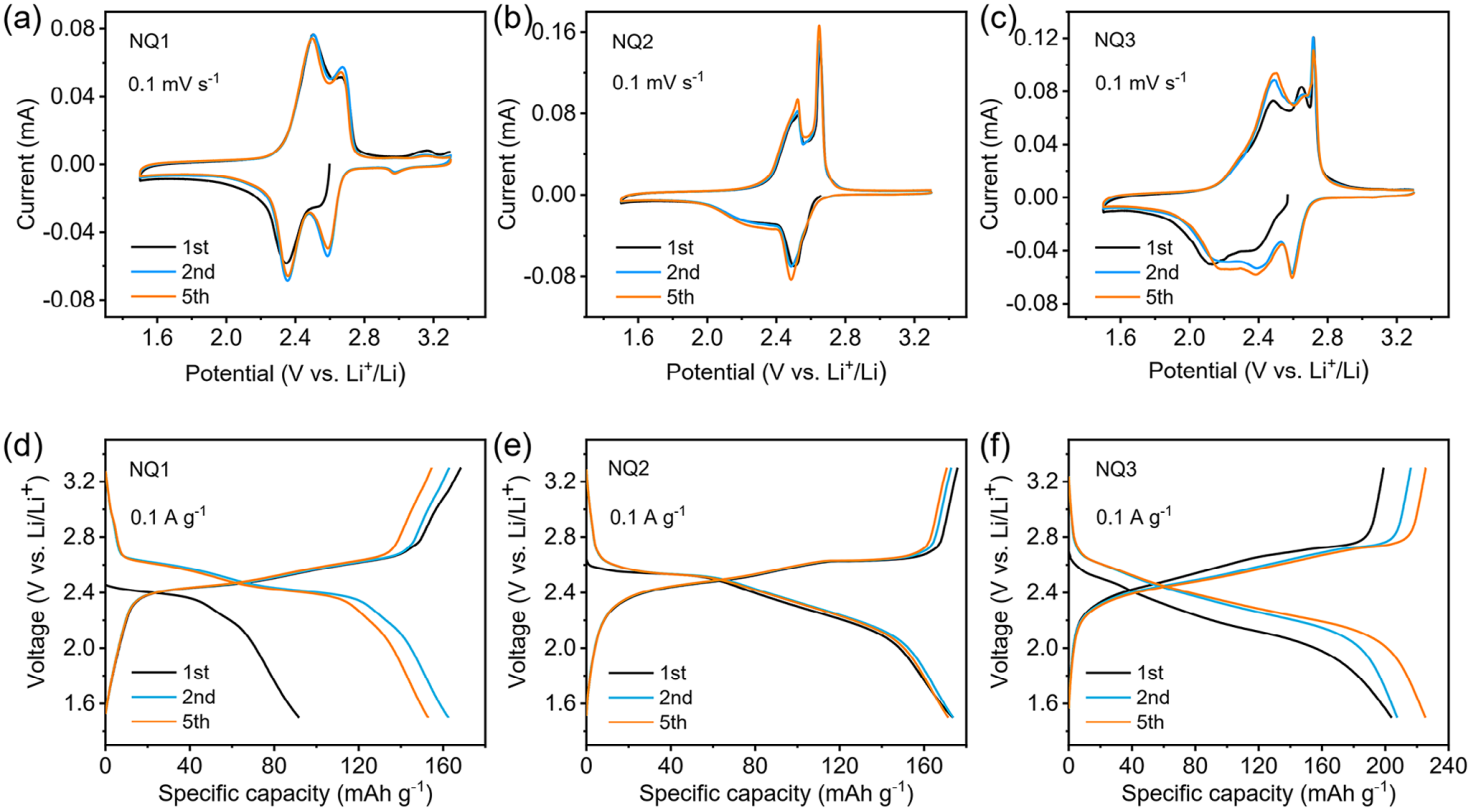
a–c) CV curves at a scan rate of 0.1 mV s^−1^ and d–f) galvanostatic charge‐discharge (GCD) profiles in selected cycles at 0.1 A g^−1^ in a voltage window of 1.5–3.3 V (vs Li^+^/Li) of NQ1 (a,d), NQ2 (b,e), and NQ3 (c, f).

**Figure** **3**a–c shows the typical cyclic voltammograms (CVs) of the half cell in the range from 3.3 to 1.5 V (vs Li/Li^+^). The CV curve of the NQ1 shows two well‐resolved redox pairs at 2.35 V/2.53 V and 2.59 V/2.68 V (vs Li/Li^+^), corresponding to its well‐known two‐step redox process (Figure 3a).^[^ ^7^, ^8^, ^9^ ^]^ NQ2 displays similar redox behavior with two comparable couples (2.29 V/2.52 V and 2.52 V/2.67 V vs Li/Li^+^), though with slightly broader peaks. Notably, NQ3 exhibits significantly broadened electrochemical features with multiple redox couples, which can be attributed to the strong electronic interactions between the three crowded naphthoquinone units in one molecule. Similar spatial effect on the redox property were also observed in other molecules electrodes.^[^ ^7^, ^8^, ^37^ ^]^

Figure 3d–f presents the galvanostatic charge/discharge (GCD) profiles of NQ1‐NQ3 at a current rate of 0.1 A g^−1^. Consistent with the CV profiles, NQ1 exhibits two distinct discharge plateaus, whereas NQ2 shows one plateau followed by a sloping region. In contrast, the discharge profile of NQ3 displays an extended sloping behavior. This gradual transition from plateaus to a sloping profile with increasing molecular size can be attributed to the enhanced electronic interference between redox centers. The average discharge voltage of three molecules is around 2.4 V. As a result of severe dissolution in electrolyte, NQ1 delivers a much lower discharge capacity of 160 mAh g^−1^ (at 2^nd^ cycle) at 0.1 A g^−1^, with an observation of obvious decay in the subsequent cycles (Figure 3d). With respect to NQ2 and NQ3, higher electrochemical activity and reversibility were observed. The GCD profiles of NQ2 at 0.1 A g^−1^ in the first 5 cycles overlap well, giving a stable capacity of 170 mAh g^−1^, corresponding to 75% of the theoretical maximum value (Figure 3e). This indicate the insufficient utilization of the redox centers in NQ2 (discussed in the vide infra). Differently, gradually increased capacity was observed for NQ3 during cycling, likely due to the activation process that provides more efficient conductive pathways as the electrode repeatedly swells and expels ions during charging and discharging cycles (vide infra). After 5 cycles, NQ3 can reach a highest capacity 224 mAh g^−1^, 95% of the theoretical value. This high practical specific capacity of NQ3 exceeds most of reported naphthoquinone based organic cathodes.

**Figure 4:**
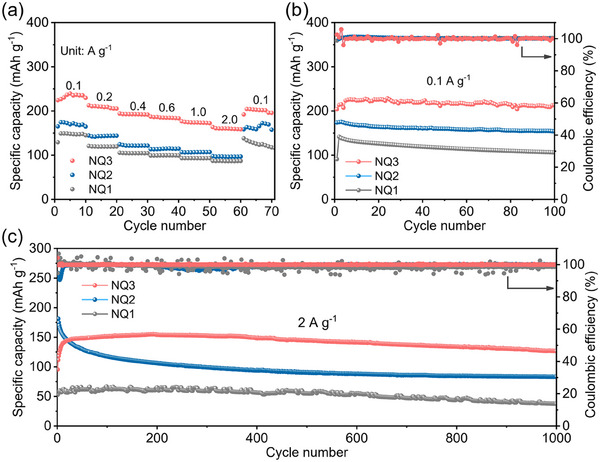
The electrochemical performance of the NQ1‐3 cathodes. a) rate performance, b,c) cycling performance at (b) 0.1 A g^−1^ and (c) 2 A g^−1^.

From the rate performance shown in **Figure** **4**a, a trend of NQ3 > NQ2 > NQ1 in terms of capacity was clearly observed under various current density. At 0.2, 0.4, 0.6, 1.0, and 2.0 A g^−1^, the NQ3 remains capacity of 208, 192, 188, 176, and 162 mAh g^−1^, respectively. When the current density was returned to 0.1 A g^−1^, the discharge capacity of NQ3 can be nearly recovered, further suggesting its excellent stability and good redox reversibility. Capacity decay with increasing current was observed for NQ1‐NQ3, and are also common for many other battery materials.^[^ ^2^, ^21^ ^]^ The reason could be attributed to kinetic factors such as ion and electron diffusion rates limit the amount of charge extracted from the electrode at higher currents.

The cycling stability were evaluated at current rate of 0.1 and 2 A g^−1^ for NQ1‐3 based cathodes. As shown in Figure 4b, NQ3 and NQ2 exhibit stable cycling performance at 0.1 A g^−1^ with the specific capacities of 212 and 154 mAh g^−1^ after 100 cycles. The high capacity retention of NQ2 (89%) and NQ3 (95%) indicate their high electrochemical stability, mainly thanks to its low solubility and superior structural stability during repeated charge/discharge cycles. Even under a high current density of 2.0 A g^−1^ after 1000 cycles, NQ3 can gives a satisfactory capacity of 127 mAh g^−1^, which is 1.5 and 3.3 times that of NQ2 and NQ1, respectively (Figure 4c). Overall, NQ3 stands out among three molecules in terms of delivered capacity during cycling test, followed by NQ2 and then NQ1. Despite NQ2 also has very low solubility and comparable *C*_thero_, its delivered capacity was much lower than NQ3. Therefore, the solubility may not the only one dominant factor accounting for the best battery performance of NQ3.

**Figure 5:**
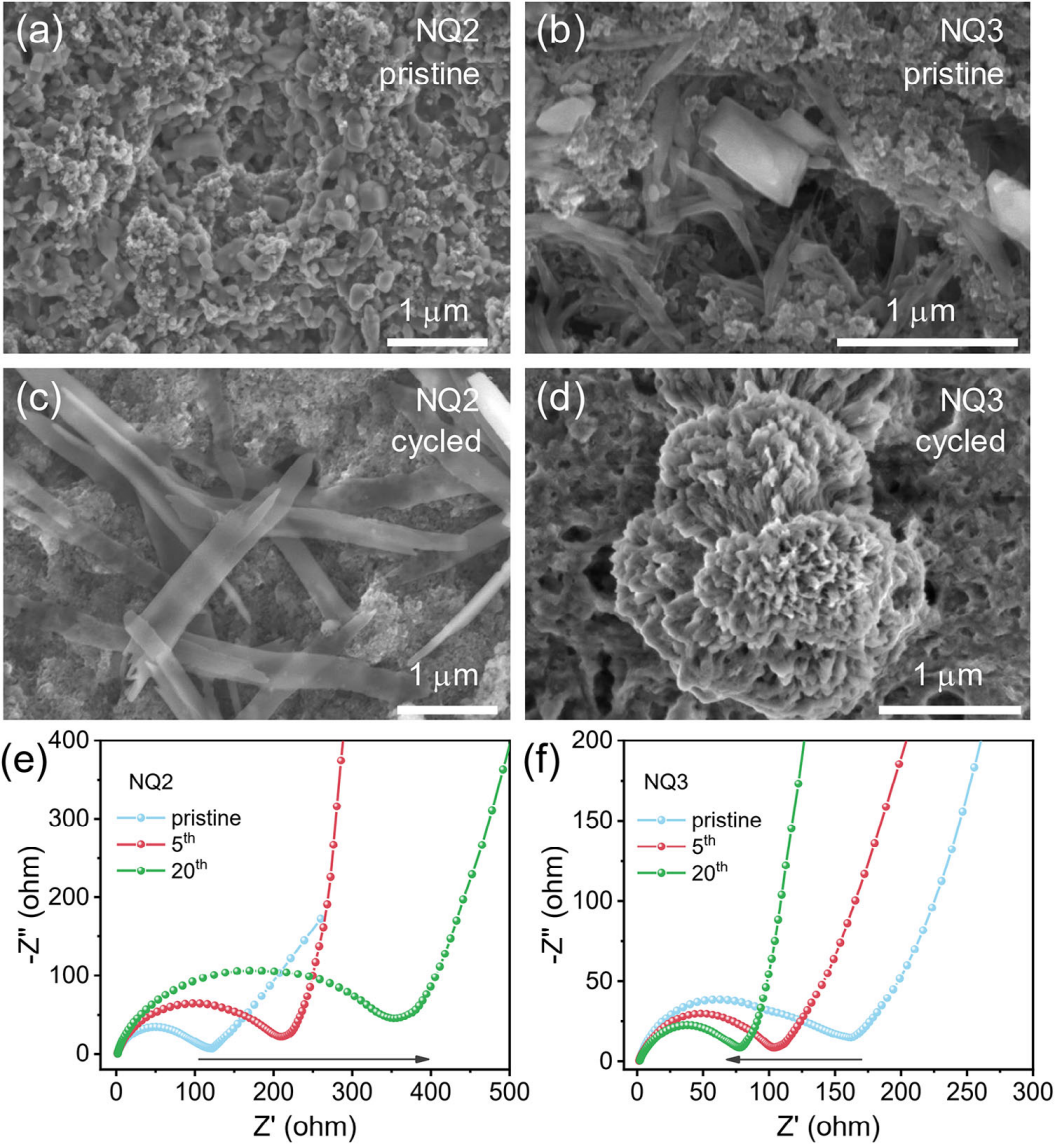
SEM images of (a, b) fresh and (c, d) cycled electrodes of NQ2 (a, c) and NQ3 (b, d). EIS curves of (e) NQ2 and (f) NQ3 electrodes in the pristine state and after 5 and 20 cycles.

To gain deep insights into the difference of battery performance between NQ2 and NQ3, the electrode morphologies before and after cycling were investigated by SEM (**Figure** **5**). In pristine electrodes, NQ2 display particle morphology; while NQ3 adopts apparent rod‐like architecture, indicating the well‐organization of the molecules as a result of stronger hydrogen bonding and π–π interaction (Figure 5a,b). Interestingly, distinct morphology evolution was observed during cycling. After cycling of NQ2, large tape‐like structure (500 nm wide, micrometers long), along with obvious heterointerfaces between active material and conductive carbon were observed (Figure 5c). These would cause poor electron transport in NQ2 electrode and insufficient utilization of the redox centers, and thus deliver lower capacity during continuous cycling. In contrast, cycling of NQ3 lead to pulverization of the pristine rod‐like aggregates, generating smaller particle size and better contact with carbon (Figure 5d). This unique feature can lead to more exposed redox‐active sites, and are beneficial for improving the electronic conductivity, which is believed to be critical factor for the better LOB performance of NQ3.

To verify the above conjecture, electrochemical impedance spectroscopy (EIS) was performed for pristine and post‐cycled batteries of NQ2 and NQ3 (Figure 5e,f). In pristine electrodes, both compounds have comparable charge transfer resistance (NQ2: *R*_ct_ = 120 Ω, NQ3: *R*_ct_ = 160 Ω). Their similar electronic conductivity for pristine NQ2 and NQ3 were also confirmed by the *I–V* measurement (Figure S13, Supporting Information). Noted that, continuous cycling of two compounds lead to completely different change of *R*_ct_. For NQ3, the *R*_ct_ gradually decrease to 102 Ω (at 5^th^ cycle), and 80 Ω (at 20^th^ cycle), signifying the enhanced electronic conductivity. However, the *R*_ct_ values of NQ2 were found to increase upon cycling, implying the poorer electron conductivity during cycling. These different trends of EIS changes are consistent with their distinct reconstruction of the electrode structures. These results can well explain the higher practical capacity and good rate performance of NQ3 than NQ2, despite they have similar theoretical capacities.

### 2.4 Redox Kinetics and Structural Evolution

**Figure 6:**
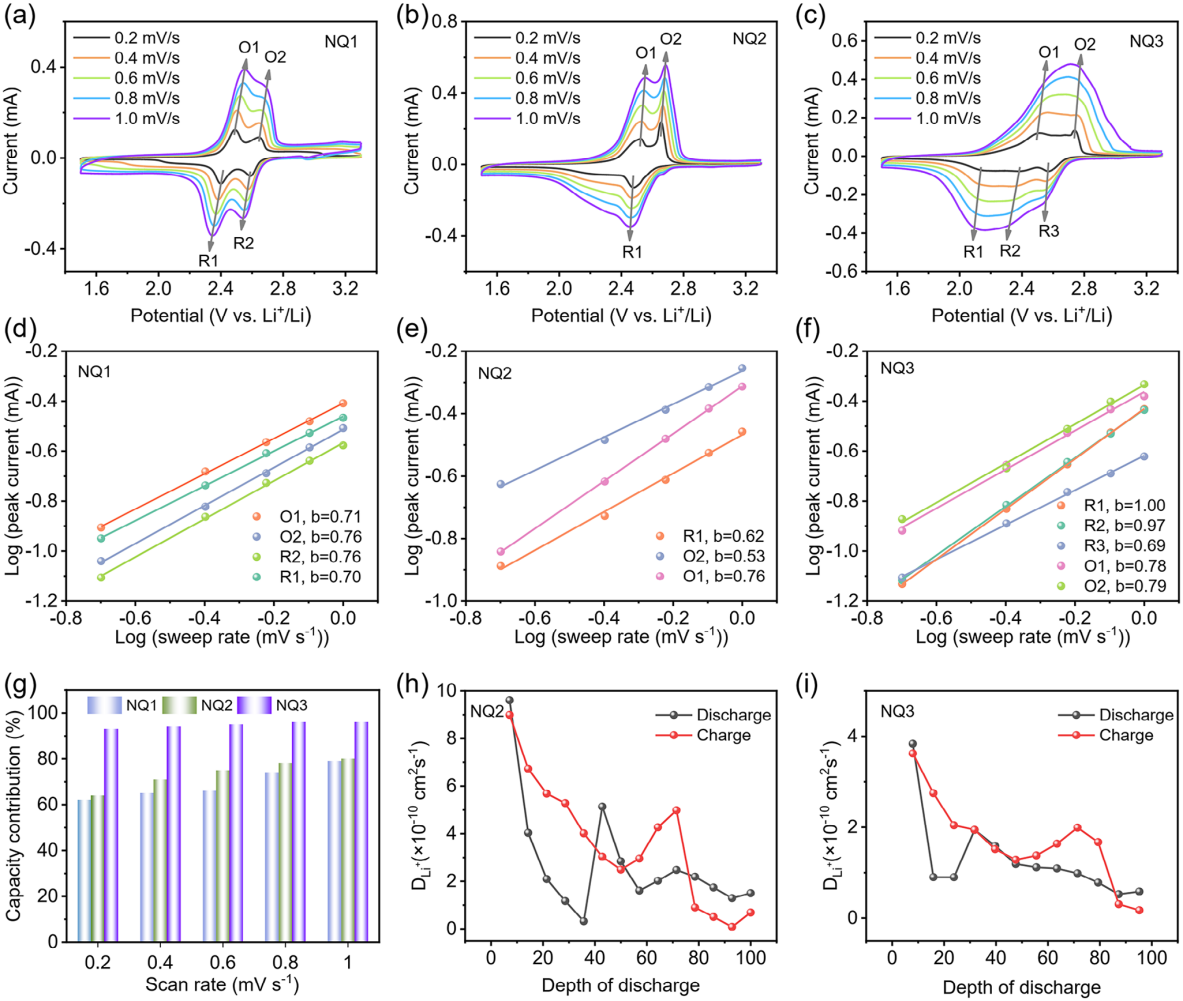
Electrochemical kinetics study of NQ1–3. a–c) CV curves of NQ1‐3 at different scan rates of 0.2–1.0 mV s^−1^. d–f) The fitted lines between log (*i*) and log (*v*) for NQ1‐3 electrodes. g) Capacitive contributions (in percentage) of NQ1‐3 at different scan rates. h,i) The Li^+^ diffusive coefficients of (h) NQ2 and (i) NQ3 calculated from the GITT profiles.

To study the redox kinetic mechanism, CVs at various scan rates from 0.2 to 1.0 mV s^−1^ were collected. With elevated scan rate, the multiple redox signals of NQ1‐NQ2 can preserve well with slight peak shifts; while the CV profile of NQ3 became broad with wide peak‐to‐peak difference (**Figure** **6**a–c). This further suggest that crowded redox centers in NQ3 interfere with each other. According to the power law,^[^ ^46^ ^]^

(1) $$i=av^{b}$$

(2) $$\log\left(i\right)=\log\left(a\right)+b\;\log\left(v\right)$$

where *i* represents the current of the CV profile, *v* is the sweep rate, and *a* and *b* are adjustable parameters. The *b* value can be obtained by the linear slope fitted between log(*i*) and log(*v*). A *b* value of 0.5 implies a diffusion‐controlled redox process; while a *b* value of 1 indicates a surface‐controlled redox reaction without diffusion limitations.

As shown in Figure 6d,e, the *b* values corresponding to the redox peaks of the NQ1 and NQ2 cathode are in the range of 0.53–0.76, indicating the synergistic effects of the surface‐controlled capacitive process and diffusion controlled redox reactions. For NQ3, the *b* values of the cathodic peaks (R1, R2, and R3) is close‐to‐unity value of 1, much higher than those for NQ1 and NQ2, suggesting that NQ3 electrodes reaction is mainly dominated by the surface‐controlled process with minor diffusion limitations (Figure 6f). By further analyzing, it was found NQ3 electrode has a larger capacitive capacity, suggesting that the NQ3 electrode has fast surface‐controlled charge‐storage kinetics. Figure 6g summarizes the contribution of the surface‐controlled capacitive process at different scan rates of NQ1‐NQ3. For NQ3, the percentage of capacitive contribution is 90% at 0.2 mV s^−1^, and the ratio reached 96% under a high scan rate of 1.0 mV s^−1^. Under same current density, the capacitive contribution to NQ1 and NQ2 are much lower. This can be rationalized from the observed morphology of the electrodes (Figure 5), where NQ3 has smaller particle size and better contact with carbon during cycling, thereby facilitating redox reactions predominantly at the surface region of the electrode. Furthermore, galvanostatic intermittent titration technique (GITT) were employed to determine the ionic diffusion (Figure 6h–i; Figure S14, Supporting Information). The Li^+^ ion diffusion coefficient (*D*_Li_^+^) for NQ2 and NQ3 were calculated to ≈ 10^−10^ cm^2^ s^−1^, that are in a comparable order of magnitude with Li^+^ diffusion in state‐of‐art *n*‐type cathodes.^[^ ^47^, ^48^, ^49^ ^]^ Consequently, the poor solubility, unique electro‐activation, high capacitive contribution and good ion diffusion coefficient enable the best LOB performance of NQ3.

**Figure 7:**
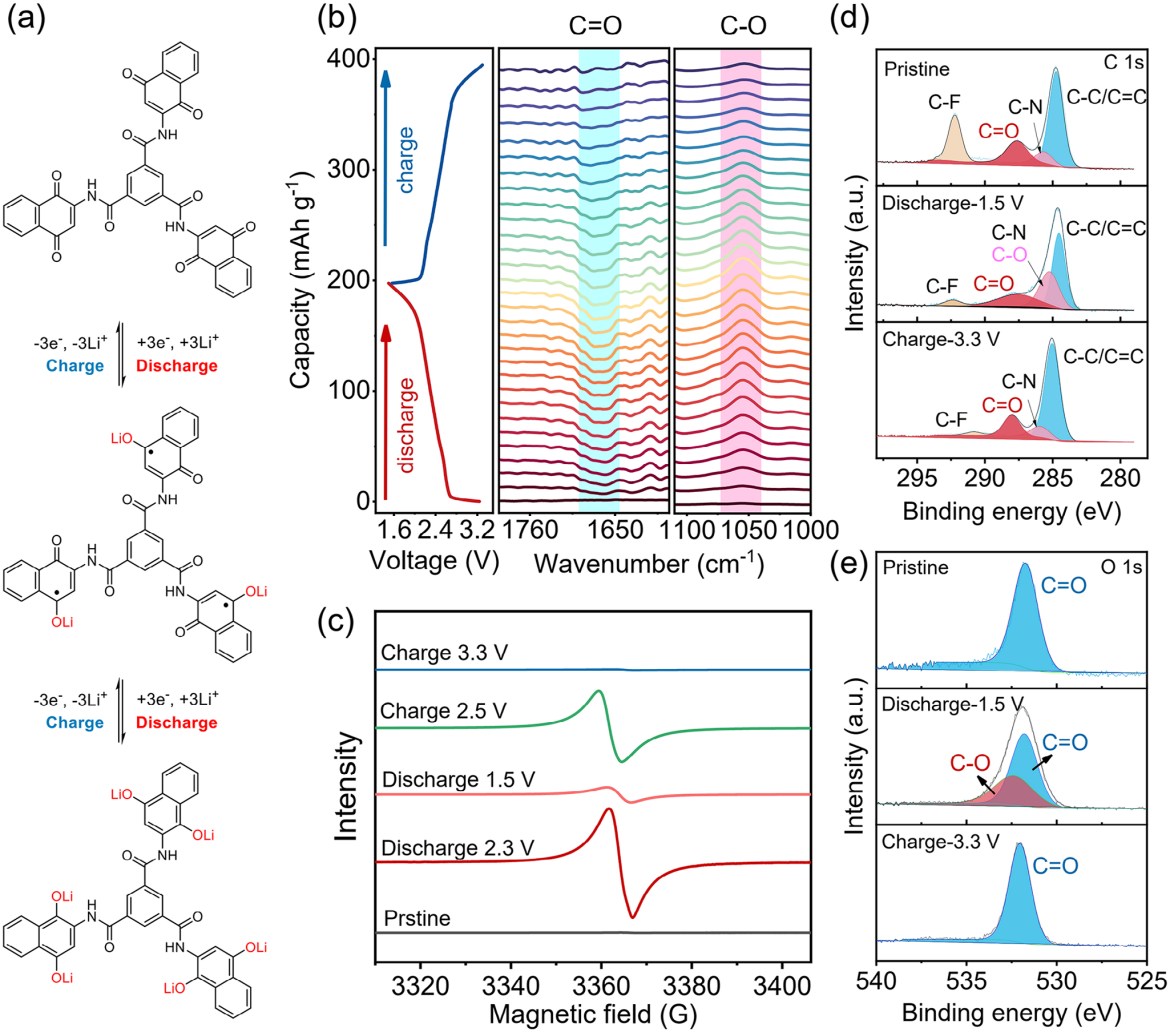
a) The electrochemical redox reaction of NQ3. b) In situ FT‐IR spectra, c) Ex situ EPR spectra, d) High‐resolution C 1s XPS spectra, e) High‐resolution O 1s XPS spectra of the NQ3 electrode during charge and discharge.

With reference to previous work on naphthoquinone, the 2e two‐step redox processes are involved in its reaction mechanism, where the redox center of NQ would undergo reversible transformation between three states: neutral NQ state, radical anion (NQ^•‐^), and dianionic species (NQ^2‒^) during charge and discharge. Meanwhile, Li^+^ ion insertion and leaving are coupled to the discharge and charge process, respectively. Therefore, the lithium storage mechanism and structure evolution of NQ3 are illustrated in **Figure** **7**a, without considering the inference between multiple redox centers. Due to the presence of three NQ unit, one NQ3 molecule would undergo six‐electron reduction during discharge process.

In order to verify this reaction mechanism, in situ FT‐IR, electron paramagnetic resonance (EPR), and X‐ray photoelectron spectroscopy (XPS) tests were performed. In the in situ FT‐IR measurements (Figure 7b), the initial state was set as the background in order to clearly present the signal change during the cycling process. The downward tendency suggests the weakening of the FT‐IR signal, while the upward indicates the formation of new functional groups. During discharging from 3.3 to 1.5 V versus Li^+^/Li, the vibration peaks of C═O group at ≈ 1665 cm^−1^ gradually weakened, while a new peak emerged at 1050 cm^−1^ that was assigned to the C─O group. During the charge process, the new peak at 1050 cm^−1^ decreased, accompanied by the recovery of the characteristic C═O group at 1665 cm^−1^. This result demonstrates the reversible transformation between the carbonyl group and C─O group.

EPR experiment was carried out to confirm the radical intermediate. As shown in Figure 7c, the EPR signal was silent in the pristine state, due to the dominant species in the neutral form. The EPR signal was strong when NQ3 was discharged to 2.3 V and almost disappeared when further discharged to 1.5 V. The result clearly confirms the presence of radical intermediate, and a stepwise two electron transfer for each NQ unit. The intensity showed a reverse trend during charge process, again reflecting the reversible electrochemical process.

The structural evolution was also verified by XPS (Figure 7d,e). Upon discharging, the C 1s signal at 287.6 eV assigned to the C═O bond decreased, along with the increased intensity of reduced C─O signal at 285.7 eV. Subsequent recharging to 3.3 V led to recovery of the C═O peak, suggesting its reliable reversibility. In the O 1s spectra, the obvious signal of C═O at 531.7 eV is shifted to C─O‐Li at 532.4 eV in the initial discharge process and partially recovered at the fully charged state. These XPS data are consistent with the FTIR data, demonstrating the relatively good stability during the charging and discharging cycles. Overall, these results clearly unveil the structural transformation of NQ3. In the discharge process, two C═O groups in each NQ unit can step‐wisely accept two electrons, forming reduced lithium enolate species via radical intermediate, In the charge process, deintercalation of Li^+^ ions occur, and carbonyl groups regain by oxidation. Similar structure evolutions of NQ1 and NQ2 are given in Figure S15 (Supporting Information).

## 3 Conclusion

In conclusion, we have successfully designed and synthesized a novel class of naphthoquinone‐derived cathode materials (NQ1‐NQ3) through a straightforward synthetic approach that strategically links one, two, and three amido‐naphthoquinone redox centers onto a benzene ring framework. The systematic increase in amide functional groups and molecular dimensions from NQ1 to NQ3 effectively addresses solubility challenges in electrolytes, reducing solubility from 90 mg mL⁻¹ for NQ1 to 0.11 mg mL⁻¹ for NQ2 and 0.05 mg mL⁻¹ for NQ3. This structural optimization translates to significantly enhanced LOB performance, with NQ3 demonstrating exceptional electrochemical characteristics. The material achieves a high specific capacity of 224 mAh g⁻¹ at 0.1 A g⁻¹ while maintaining 95% capacity retention over 100 cycles. Remarkably, NQ3 exhibits outstanding long‐term cyclability at elevated current densities, sustaining 127 mAh g⁻¹ after 1000 cycles at 2.0 A g⁻¹. Notably, post‐cycling characterization revealed an intriguing morphological evolution of NQ3 particles, showing size reduction and enhanced carbon contact that likely contributes to its superior stability. These results demonstrate that strategic molecular engineering to strengthen intermolecular hydrogen bonding networks and π–π stacking interactions presents an effective pathway for mitigating dissolution issues in organic electrode materials.

[CCDC 2445772 contains the supplementary crystallographic data for this paper. These data can be obtained free of charge from The Cambridge Crystallographic Data Centre via www.ccdc.cam.ac.uk/data_request/cif.]

## Conflict of Interest

The authors declare no conflict of interest.

## Supporting information



Supporting Information

## Data Availability

The data that support the findings of this study are available from the corresponding author upon reasonable request.
