# Supplementary material for: Rational Molecular Engineering of Amidonaphthoquinone Cathodes: Precise Hydrogen Bond and Size Control for High‐Performance Lithium Organic Batteries
Source: Adv Sci (Weinh). 2025 May 23;12(30):e05936. doi: 10.1002/advs.202505936 (PMC12376649; doi:10.1002/advs.202505936)
Supplement: Supplementary file 1 — Supporting Information [file ADVS-12-e05936-s001.docx]

*Supporting Information*

**Rational Molecular Engineering of Amidonaphthoquinone Cathodes: Precise Hydrogen Bond and Size Control for High-Performance Lithium Organic Batteries**

*Qianglong Chen, Fangfang Xing, Jia Cai, Xiujuan Wang, Xiaoming He**

^*^To whom correspondence should be addressed

Key Laboratory of Applied Surface and Colloid Chemistry (Ministry of Education), School of Chemistry and Chemical Engineering, Shaanxi Normal University, Xi’an 710119, P.R. China

*Corresponding Author Email: xmhe@snnu.edu.cn

**1. Materials**

*O*-methylhydroxylamine hydrochloride, benzoyl chloride, 1,4-benzenedicarbonyl dichloride, 1,3,5-benzenetricarbonyl trichloride and 1,4-naphthoquinone were purchased from Sinopharm Chemical Reagent Co., Adamas or Alfa Aesar. Poly(vinylidene fluoride) (PVDF, M_w_ = 534000), N-methylpyrrolidone (NMP), Ketjen Black (ECP-600J) were obtained from Shenzhen Kejing Zhida Technology Co., Ltd. The lithium chip, CR2032 battery case, 1.0 M lithium bistrifluoromethanesulfonimide (LiTFSI) in DOL/DME (1:1, v/v), aluminum foil and separator (Celgard2400) were purchased from DoDoChem. PVDF and Ketjen Black (ECP-600J) were dried at 80 ^o^C for 12 h under vacuum oven before use. All other reagents or solvents were used without further purification, unless indicated otherwise.

**2. Instruments**

^1^H NMR and ^13^C NMR spectra were obtained on 600 MHz BRUKER spectrometer. Mass spectrometry data were collected on a Bruker maxis UHR-TOF mass spectrometer. IR spectra were obtained on a PE-Frontier instrument. Cyclic voltammetry (CV) analyses were performed on a Shanghai Chenhua CHI760E instrument, with a polished glassy carbon electrode as the working electrode, a Pt-wire as counter electrode, and 0.01 M Ag/AgCl (3 M KCl) as a reference electrode. Ultraviolet-visible (UV-vis) absorption spectra were obtained on Cary 3500 spectrometer. Scanning electron microscope (SEM) images were recorded using a Hitachi SU8220 system. The morphology and structure of the obtained samples were identified using powder X-ray diffraction (XRD, Bruker D8 Advance with Cu-Kα radiation). Thermogravimetric analysis (TGA) was performed under nitrogen atmosphere from 25 to 800 ℃ with a heating rate of 10 ℃ min^-1^. The X-ray photoelectron spectra (XPS) experiments were carried out on a PHI-5400 electron spectrometer. Theoretical calculations were carried out at the B3LYP/6-31G (d, p) level by using the GAUSSIAN 09 suite of programs.^S1^

**3. Synthesis**

**Synthesis of N-Methoxybenzamide (S1).** To a solution of K_2_CO_3_ (22.2 g, 160 mmol) in a mixture of CH_2_Cl_2_/H_2_O (240 mL, 2:1, v/v) was added *O*-methylhydroxylamine hydrochloride (10.1 g, 120 mmol). The resulting solution was cooled to 0 ºC, followed by dropwise addition of a solution of Benzoyl chloride (11.2 g, 80 mmol) in CH_2_Cl_2_ (40 mL). The reaction mixture was warmed to room temperature and stirred overnight. The organic layers were separated, dried over anhydrous MgSO_4_, filtered and concentrated. The products were obtained as pure solids without need for further purification (Yield: 78 %). ^1^H NMR (600 MHz, d6-DMSO): *δ* (ppm) = 11.74 (s, 1H), 7.75 (d, *J* = 7.8 Hz, 2H), 7.55 (t, *J* = 7.8 Hz, 1H), 7.47 (t, *J* = 7.8 Hz, 2H).

**Synthesis of N1,N4-dimethoxybenzene-1,4-dicarboxamide (S2).** To a solution of K_2_CO_3_ (22.2 g, 160 mmol) in a mixture of CH_2_Cl_2_/H_2_O (240 mL, 2:1, v/v) was added *O*-methylhydroxylamine hydrochloride (10.1 g, 120 mmol). The resulting solution was cooled to 0 ºC, followed by dropwise addition of a solution of 1,4-benzenedicarbonyl dichloride (8.12 g, 40 mmol) in CH_2_Cl_2_ (40 mL). The reaction mixture was warmed to room temperature and stirred overnight. The resulting precipitate was collected by filtration, washed with H_2_O and CH_2_Cl_2_, and dried to yield the product as white solid (Yield: 8.4 g, 94 %). ^1^H NMR (600 MHz, d6-DMSO): *δ* (ppm) = 11.87 (s, 2H), 7.80 (s, 4H), 3.70 (s, 6H).

**Synthesis of N1, N3, N5-trimethoxybenzene-1,3,5-tricarboxamide (S3).** To a solution of K_2_CO_3_ (22.2 g, 160 mmol) in a mixture of CH_2_Cl_2_/H_2_O (240 mL, 2:1, v/v) was added *O*-methylhydroxylamine hydrochloride (10.1 g, 120 mmol). The resulting solution was cooled to 0 ºC, followed by dropwise addition of a solution of 1,3,5-benzenetricarbonyl trichloride (7.1 g, 26.7 mmol) in CH_2_Cl_2_ (40 mL). The reaction mixture was warmed to room temperature and stirred overnight. The resulting precipitate was collected by filtration, washed with H_2_O and CH_2_Cl_2_, and dried to yield the product as white solid (Yield: 7.0 g, 88 %). ^1^H NMR (600 MHz, d6-DMSO): *δ* (ppm) = 12.02 (s, 3H), 8.27 (s, 3H), 3.73 (s, 9H).

**Synthesis of NQ1.** A mixture of 1,4-naphthoquinone (4.4 g, 28 mol), **S1** (3.8 g, 25 mmol), *i*Pr_2_NEt (9 mL, 50 mmol) in CH_3_CN (200 mL) was stirred in at 70 ºC for 12 hours. After removal of volatiles under vacuum, the residues were purified on silica flash column with AcOEt/hexane (1:10) as eluents to obtain the final product as brown solid (Yield: 85 %). ^1^H NMR (600 MHz, CDCl_3_): *δ* (ppm) = 9.21 (s, 1H), 8.15 (t, *J* = 7.8 Hz, 2H), 8.03 (s, 1H), 7.95 (d, *J* = 7.8 Hz, 2H), 7.81 (t, *J* = 7.8 Hz, 1H), 7.75 (t, *J* = 7.8 Hz, 1H), 7.62 (t, *J* = 7.8 Hz, 1H), 7.55 (t, *J* = 7.8 Hz, 2H). ^13^C NMR (150 MHz, CDCl_3_): *δ* (ppm) = 185.15, 181.23, 165.82, 140.13, 135.07, 133.32, 133.27, 132.97, 132.30, 130.00, 129.07, 127.36, 126.72, 126.48, 117.31. HR-MS: m/z calculated for C_17_H_12_NO_3_^+^ [M+H]^+^ 278.0812, found 278.0814.

**Synthesis of NQ2.** A mixture of 1,4-naphthoquinone (15.8 g, 100 mol), **S2** (5.6 g, 25 mmol), *i*Pr2NEt (26 mL, 150 mmol) in DMF (250 mL) was stirred in at 70 ºC for 60 hours under N_2_. The resulting precipitate was collected by hot filtration, washed with DMF, EtOH and THF. The products were dried *in vacuo* for 24 h. Yield: 6.4 g, 54 %. ^1^H NMR (600 MHz, CDCl_3_/CF_3_COOD (9:1, v/v)): *δ* (ppm) = 9.46 (s, 2H), 8.27 (b, 2H), 8.23 (b, 2H), 8.14 (b, 4H), 8.11 (s, 2H), 7.92-7.85 (m, 4H). ^13^C NMR (150 MHz, CDCl_3_/CF_3_COOD (9:1, v/v)): *δ* (ppm) = 188.43, 180.25, 167.11, 140.53, 136.68, 135.91, 134.88, 131.64, 129.71, 128.51, 127.57, 127.30, 117.80. HR-MS: m/z calculated for C_28_H_17_N_2_O_6_^+^ [M+H]^+^ 477.1081, found 477.1077.

**Synthesis of NQ3.** A mixture of 1,4-naphthoquinone (15.8 g, 100 mol), **S3** (4.95 g, 16.7 mmol), *i*Pr_2_NEt (26 mL, 150 mmol) in DMF (250 mL) was stirred in at 70 ºC for 60 hours under N_2_. The resulting precipitate was collected by hot filtration, washed with DMF, EtOH and THF. The products were dried *in vacuo* for 24 h. Yield: 5.6 g, 50 %. ^1^H NMR (600 MHz, CDCl_3_/CF_3_COOD (9:1, v/v)): *δ* (ppm) = 9.60 (s, 3H), 8.81 (s, 3H), 8.22-8.18 (m, 6H), 8.12 (s, 3H), 7.92-7.85 (m, 6H). ^13^C NMR (150 MHz, CDCl_3_/CF_3_COOD (9:1, v/v)): *δ* (ppm) = 188.96, 180.38, 166.40, 140.84, 135.96, 135.08, 134.59, 131.47, 131.39, 129.70, 127.61, 127.26, 118.38.

**4.** **Battery Fabrication and Testing**

The working electrodes were prepared by mixing the NQ1 or NQ2 or NQ3, Ketjen Black (ECP-600J) and PVDF with a mass ratio of 6/3/1 (unless other noted) using NMP as dispersion solvent. Then the slurry was coated on the aluminum foil with a diameter of 9 mm and the mass loading of the active materials was *ca.* 0.6-0.8 mg cm^-2^. The fully prepared electrodes were then dried at 80 °C overnight in a vacuum oven in order to remove any residual solvent. Assembly of the CR2032-type coin-type half cells were carried out in argon-filled glove box (<0.01 ppm of oxygen and water), with lithium metal as the anode, polypropylene separator film (Celgard2400) as the separator, and 1.0 M lithium bistrifluoromethanesulfonimide (LiTFSI) in DOL/DME (1:1, v/v) as the electrolyte. The CV and EIS tests were performed an CHI760E electrochemical working station, and the charge-discharge curves were conducted on LAND CT2001A at 30 ℃, unless otherwise noted.

**5. Calculations of the Electrochemical Metrics**

Theoretical capacity (*C*theor, mAh g^–1^) was calculated according to the equation (1):

$C_{theor}=\frac{nF}{3.6 M}$ (1)

where *n* is the number of electrons transferred per molecules, *F* is the Faraday’s constant (96484 C mol^-1^), *M* is molecular weight of the molecules.

The *b*-value and capacitive contribution at a particular potential were determined as follows:

The relationship between scan rate (υ, mV s^-1^) in a CV and the corresponding cathodic or anodic peak current (*i*_p_, A g^-1^) is shown in equation (2).^S2^ The *b*-value was the slope of the log(*v*)-log(*i*_p_) plots according to equation (3).

$i_{p}=av^{b}$ (2)

$\log\left( i_{p} \right)=\log(a)+b\log(v)$ (3)

where *a* and *b* are adjustable parameters.

Moreover, the relationship between the current at a particular potential (*i*(V), A g^-1^) and the scan rate (υ, mV s^-1^) is shown in equation (4).^S3^ Solving for the values of *k*_1_ and *k*_2_ at each potential, we can obtain the percentage of capacitive contribution the total current (*k*_2_*v*/*i*(V)).

$i\left( V \right)= k_{1}v^{1/2}+k_{2}v$ (4)

**6. Electronic conductivity calculation**

Electronic conductivity (σ, S cm^-1^) was calculated according to the equation (5):

$\sigma=\frac{L}{R S}$ (5)

where *L* (cm) is the thickness of the sheet, *R* (Ω) is the impedance of the sheet active material, *S* (cm^2^) is the surface area of the sheet. Conductivity was measured by linear scanning voltammetry (LSV). The polymer powders were pressed into sheets and then sandwiched between two electrodes for LSV test at 100 mV s^-1^.

**7. Calculation of apparent ion diffusion coefficients (*D*_app_, cm^2^ s^-1^) from GITT**

The apparent diffusion coefficients (*D*_app_) are calculated by the following equation: ^S4^

$D_{app}= \frac{4}{\pi\tau}\left( \frac{V_{m}m_{B}}{SM_{m}} \right)^{2}\left( \frac{{\Delta E}_{s}}{{\Delta E}_{t}} \right)^{2}$ (6)

where *D*_app_ is the apparent ion diffusion coefficient (cm^2^ s^-1^), τ is the the duration of the current pulse (s), *V*_m_ is the molar volume of the active material (cm^3^ mol^-1^), *m*_B_ is the mass of the active material in the electrolyte (g), *M*_m_ is the molar mass of active material (g mol^-1^), S is the contact surface area (cm^2^) between electrode and electrolyte, *ΔE*_s_ is the equilibrium potential change induced by current pulse, *ΔE*_t_ is the potential variation during the constant current pulse.

8. Supplementary Tables and Figures.

**Table S1**. Crystal data and structure refinement for NQ1.

| Compounds | NQ1 |
| --- | --- |
| Empirical formula | C_17_H_11_NO_3_ |
| Formula weight | 277.27 |
| Temperature/K | 200.00 |
| Crystal system | orthorhombic |
| Space group | Pbca |
| a/Å | 12.7680(5) |
| b/Å | 12.5682(4) |
| c/Å | 16.5635(7) |
| α/° | 90 |
| β/° | 90 |
| γ/° | 90 |
| Volume/ Å^3^ | 2657.96(17) |
| Z | 8 |
| ρ_calc_ g/cm^3^ | 1.386 |
| μ / mm^-1^ | 0.096 |
| F(000) | 1152.0 |
| Crystal size/ mm^3^ | 0.3 × 0.2 × 0.1 |
| Radiation | MoKα (λ = 0.71073) |
| 2θ range for data collection/° | 4.918 to 52.774 |
| Index ranges | -15≤ *h* ≤ 15, -15 ≤ *k* ≤ 15,  -20 ≤ *l* ≤ 20 |
| Reflections collected | 50112 |
| Independent reflections | 2715 [R_int_ = 0.0552, R_sigma_ = 0.0179] |
| Data / restraints / parameters | 2715 / 0/ 194 |
| Goodness-of-fit on F^2^ | 1.053 |
| Final R indexes [I > 2ϭ(I)] | R_1_ = 0.0344, wR_2_ = 0.0859 |
| Final R indexes (all data) | R_1_ = 0.0426, wR_2_ = 0.0912 |
| Largest diff. peak and hole/ eÅ^-3^ | 0.22 and -0.17 |
| CCDC | 2445772 |


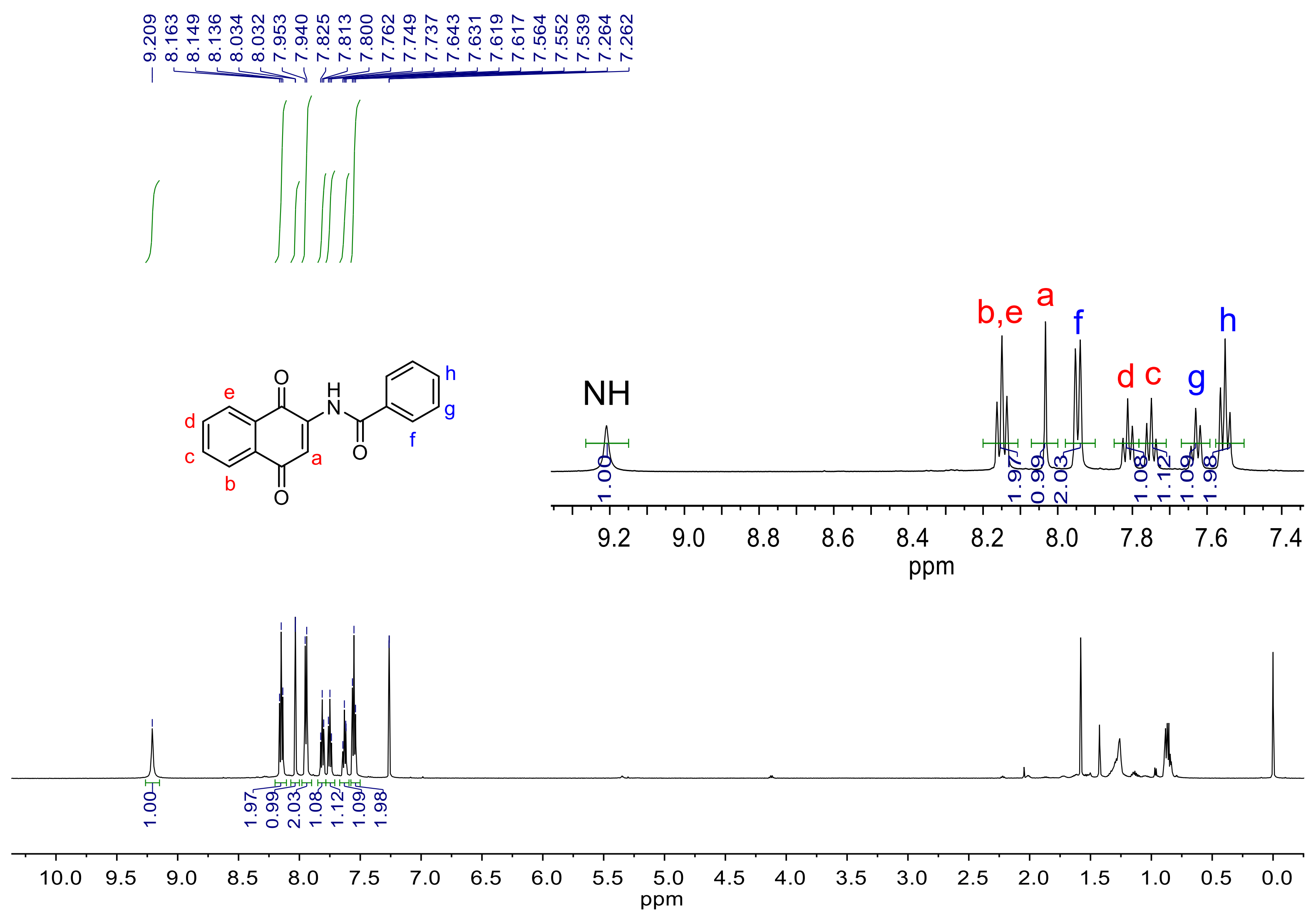


**Figure S1**. ^1^H NMR spectrum of NQ1 in CDCl_3_ at 298 K.


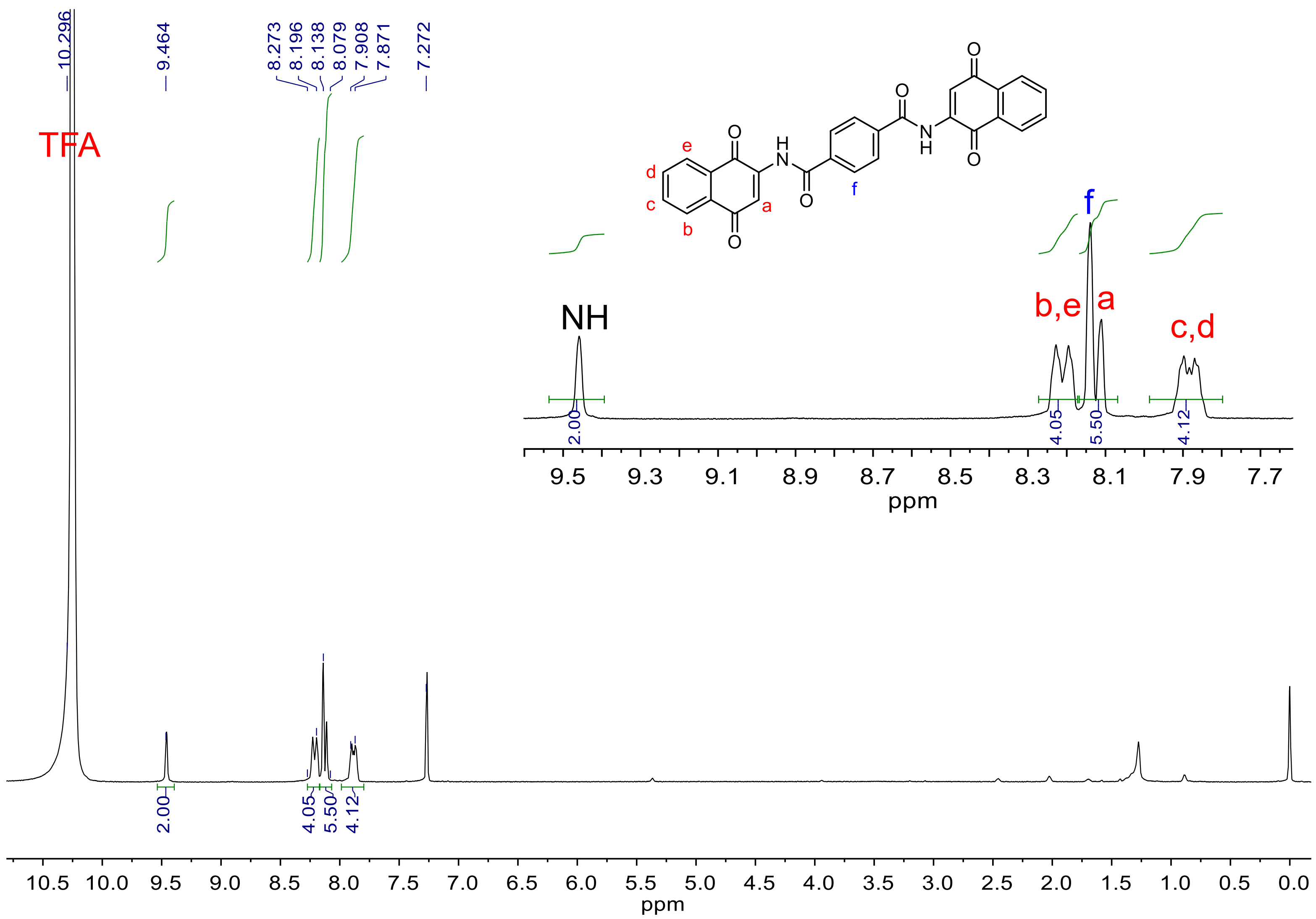


**Figure S2**. ^1^H NMR spectrum of NQ2 in CDCl_3_/CF_3_COOD (9:1, v/v) at 298 K.


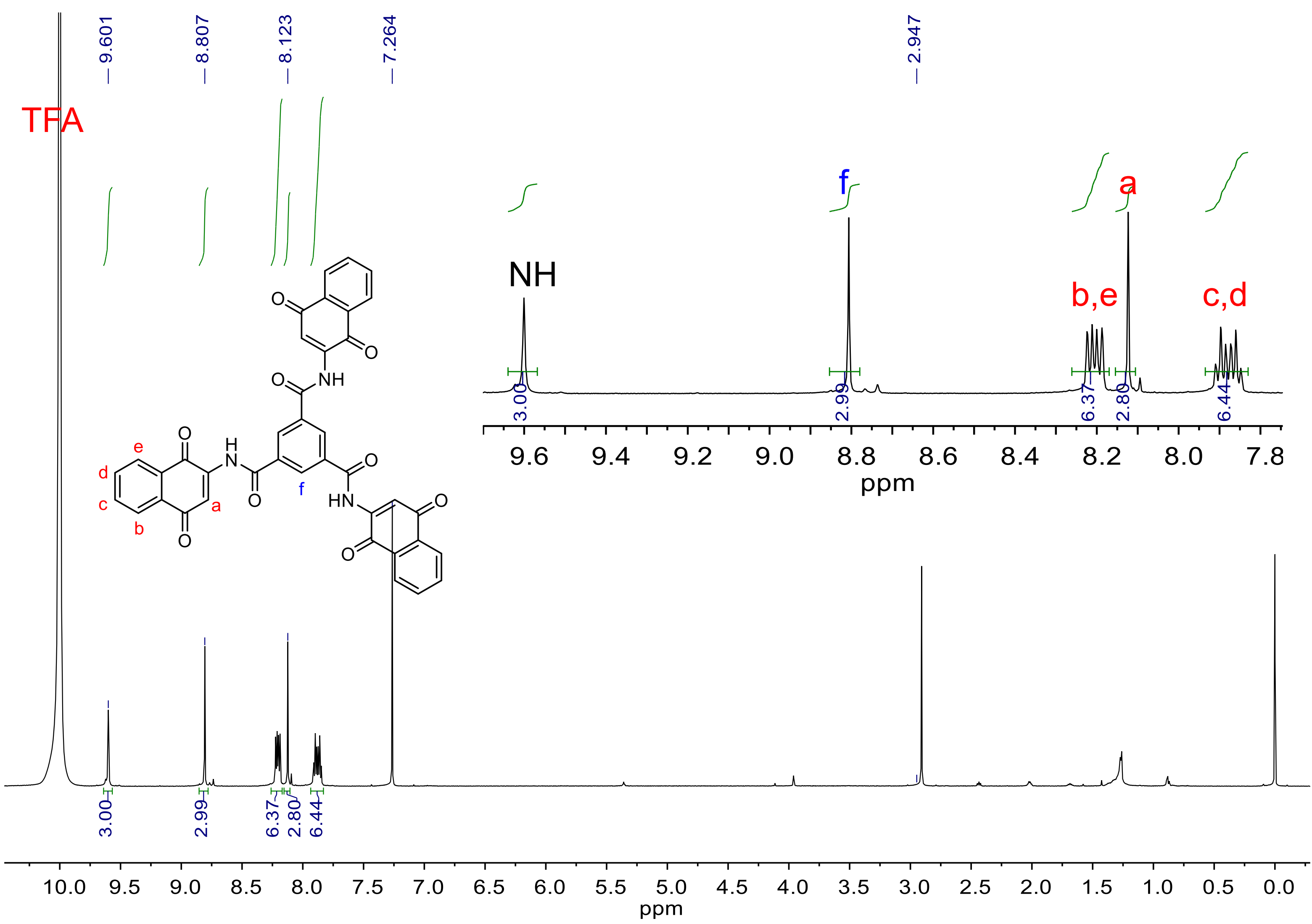


**Figure S3**. ^1^H NMR spectrum of NQ3 in CDCl_3_/CF_3_COOD (9:1, v/v) at 298 K.


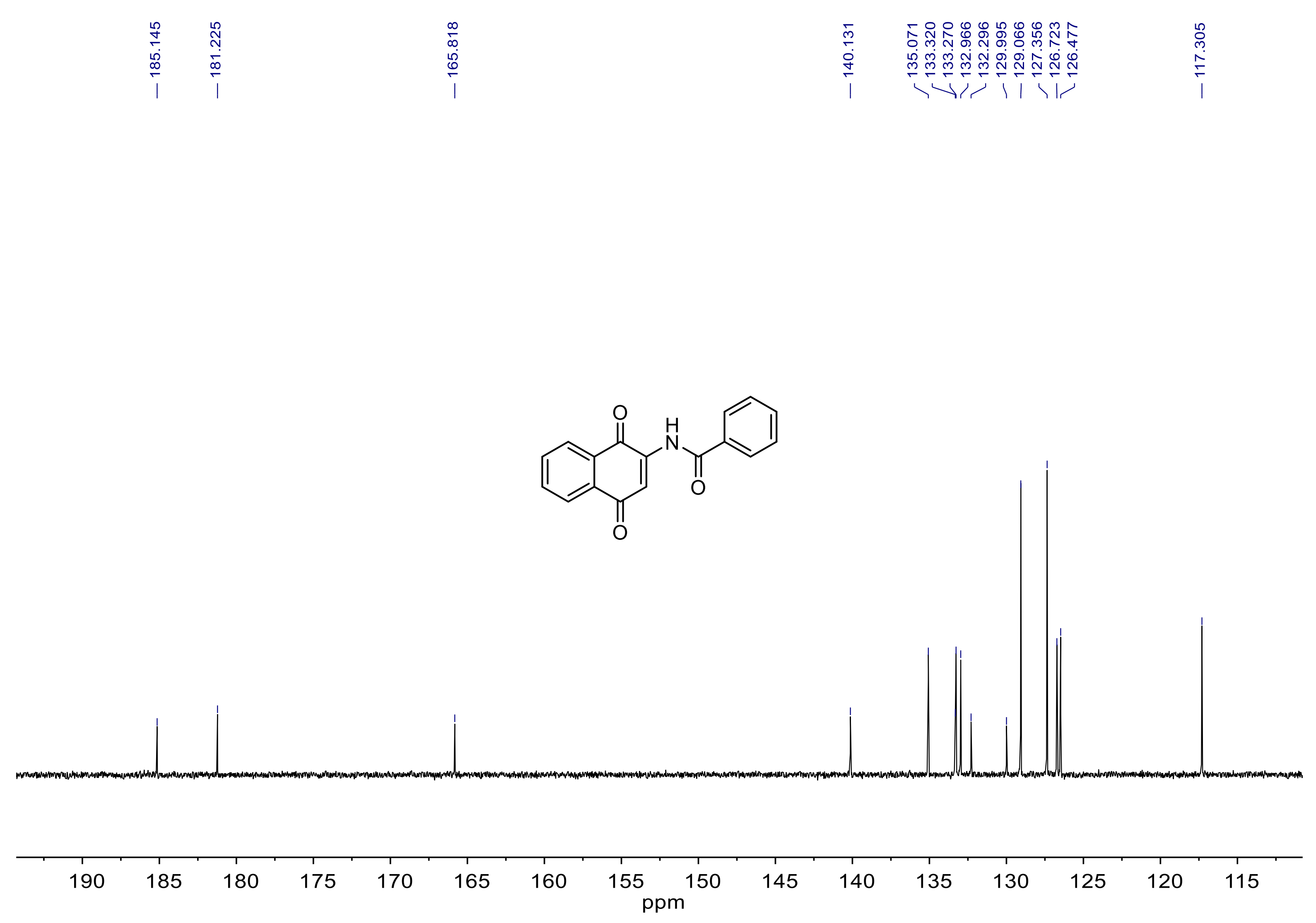


**Figure S4**. ^13^C NMR spectrum of NQ1 in CDCl_3_.


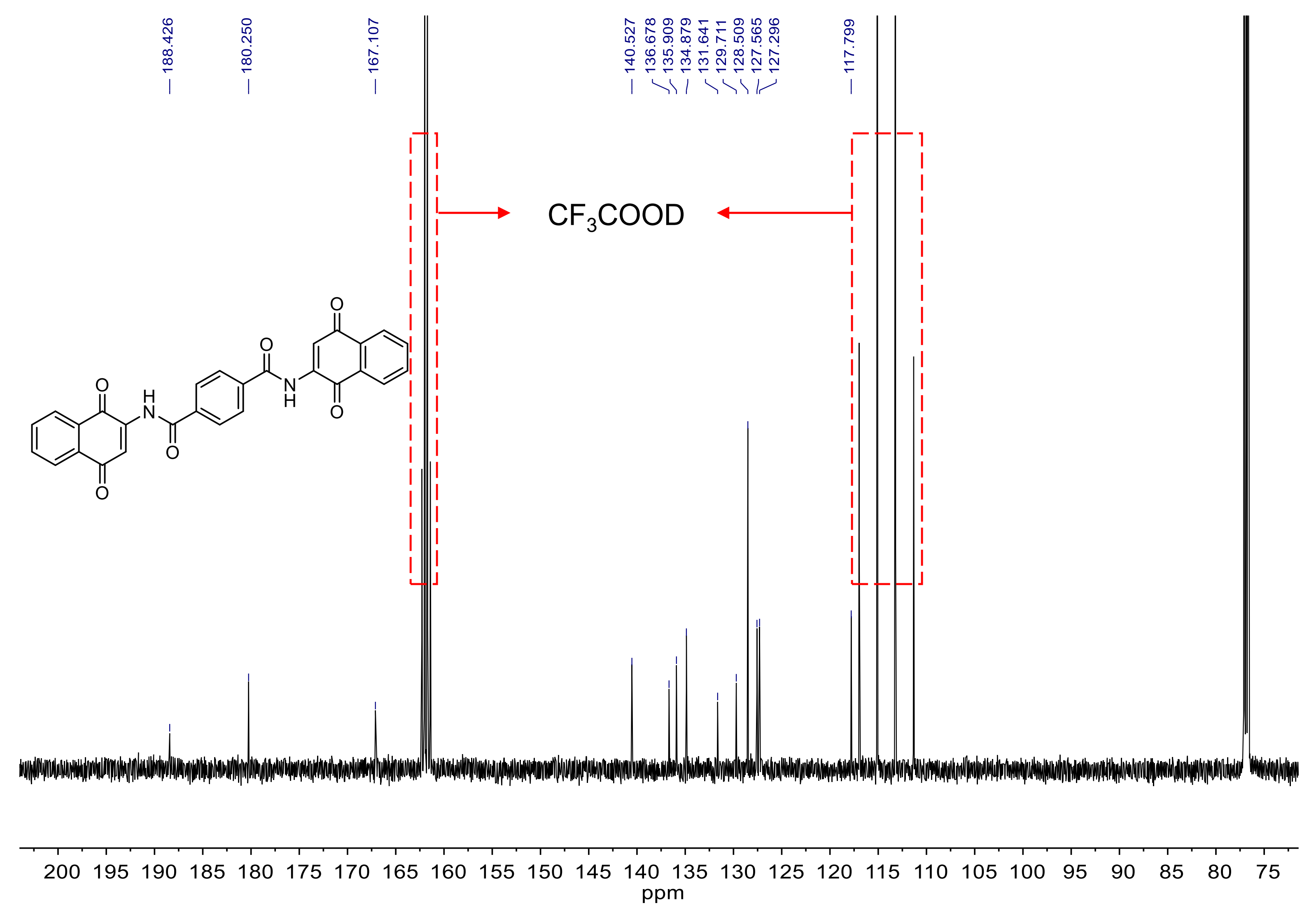


**Figure S5**. ^13^C NMR spectrum of NQ2 in CDCl_3_/CF_3_COOD (9:1, v/v).


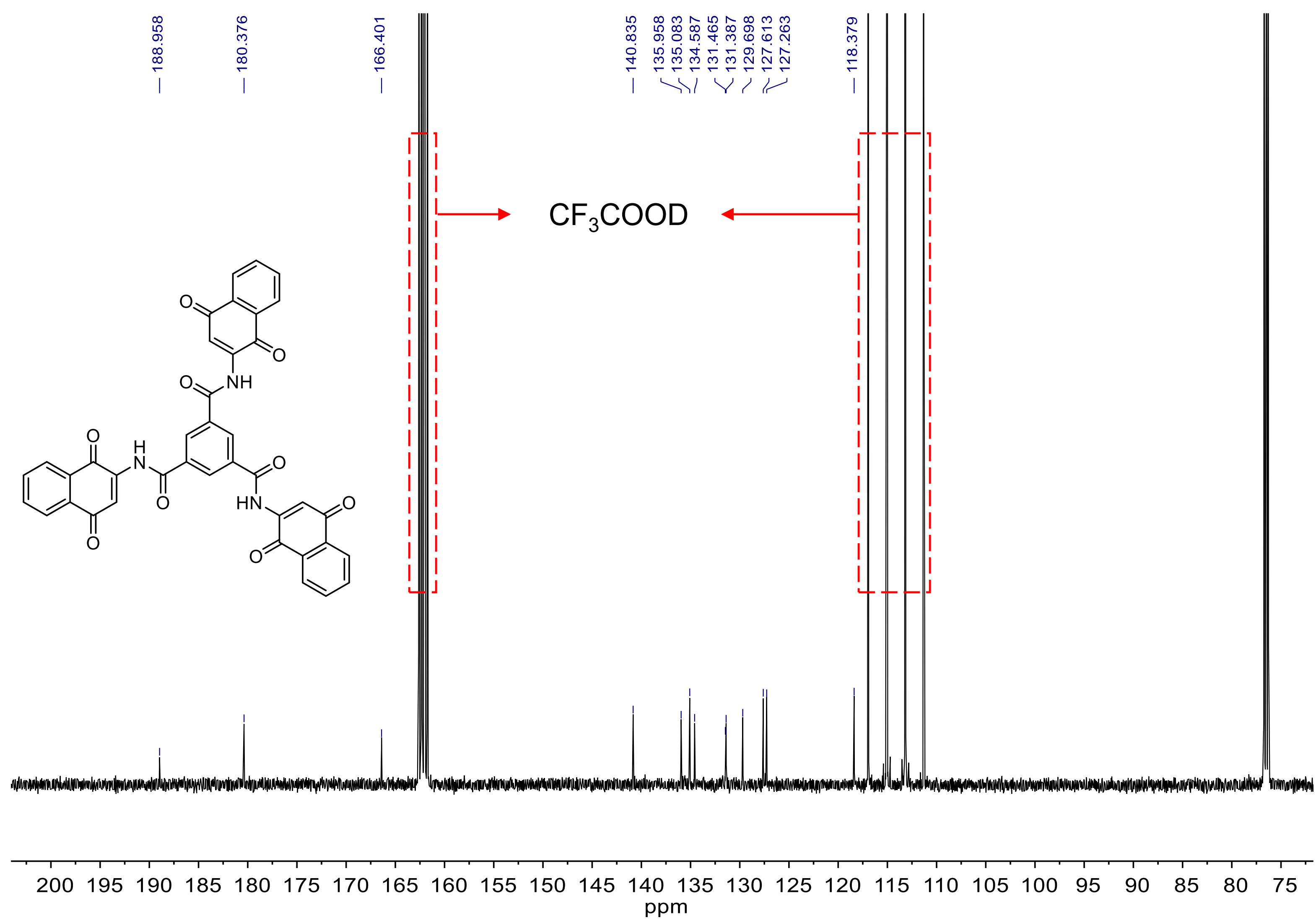


**Figure S6**. ^13^C NMR spectrum of NQ3 in CDCl_3_/CF_3_COOD (9:1, v/v).


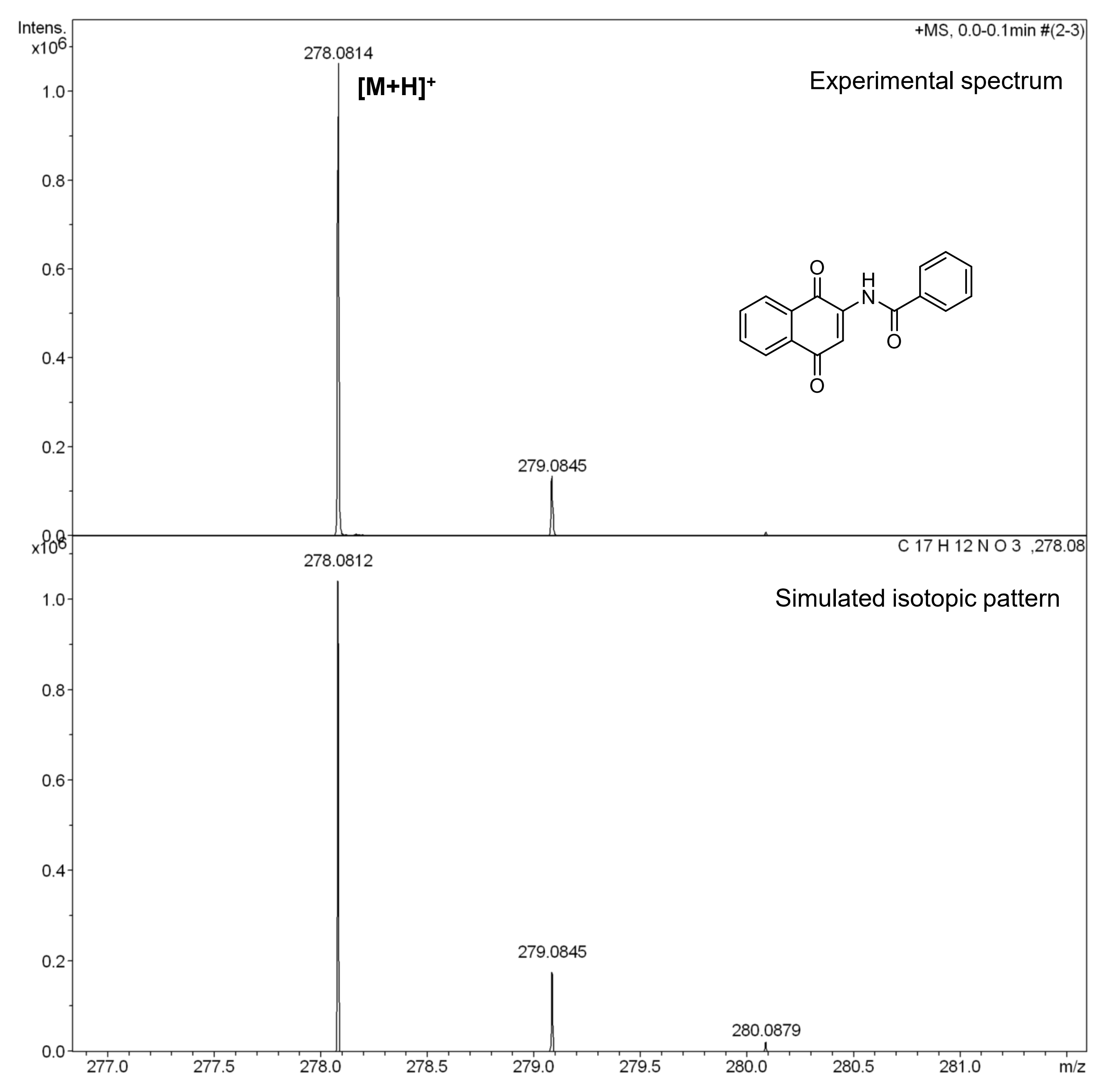


**Figure S7**. HR-MS spectrum of NQ1.


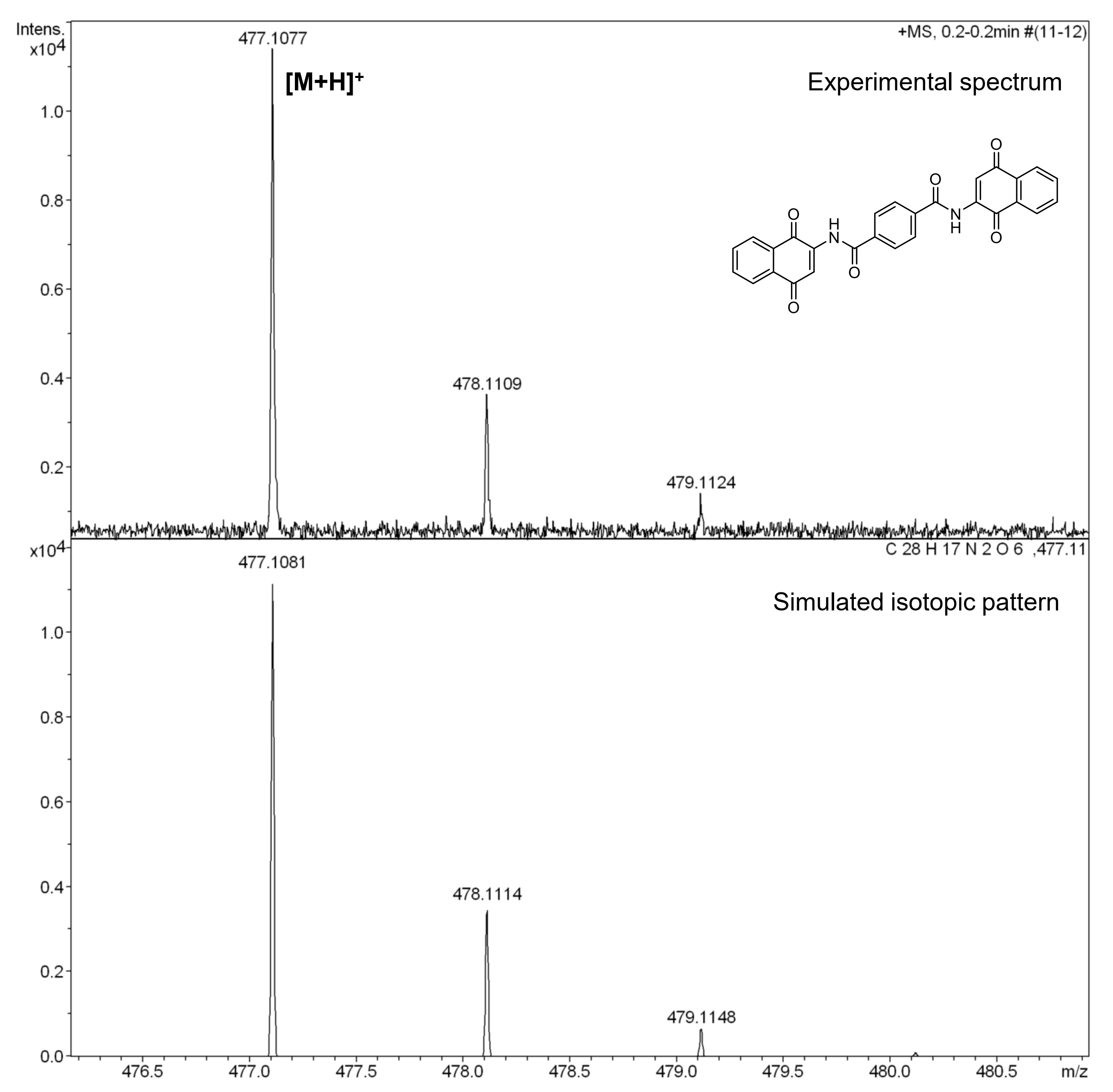


**Figure S8**. HR-MS spectrum of NQ2.

**Figure S9**. Thermal gravimetric analysis (TGA) profiles of NQ1, NQ2 and NQ3.


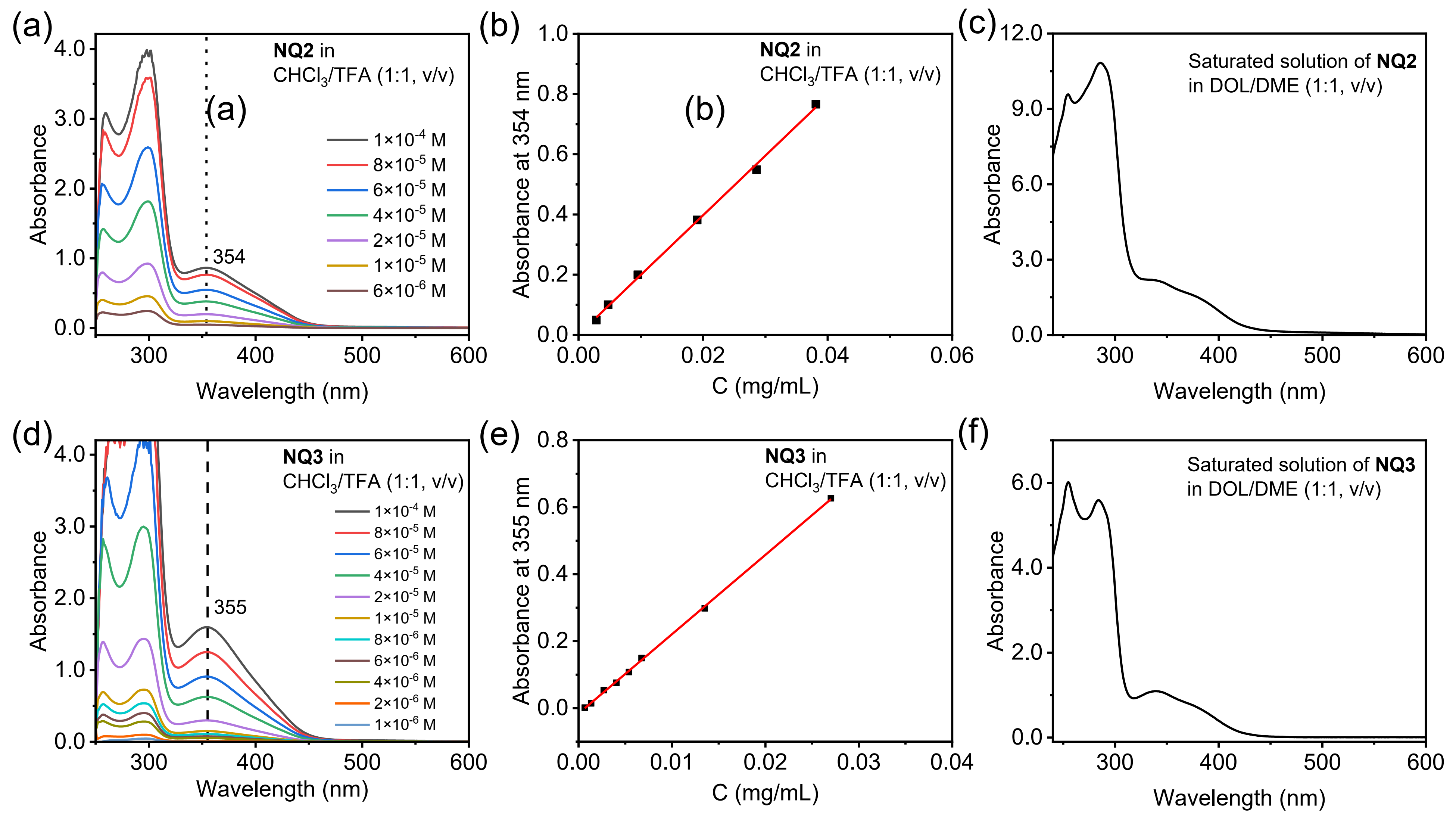


**Figure S10.** (a) UV-vis spectra of NQ2 in CHCl_3_/TFA (1:1, v/v) at different concentrations and (b) fitting curve of absorbance at 354 nm with concentration. (c) UV-vis spectrum of NQ2 in saturated DOL/DME (1:1, v/v) solution. (d) UV spectra of NQ3 in CHCl_3_/TFA (1:1, v/v) at different concentrations and (e) fitting curve of absorbance at 355 nm with concentration. (f) UV-vis spectrum of NQ3 in saturated DOL/DME (1:1, v/v) solution.


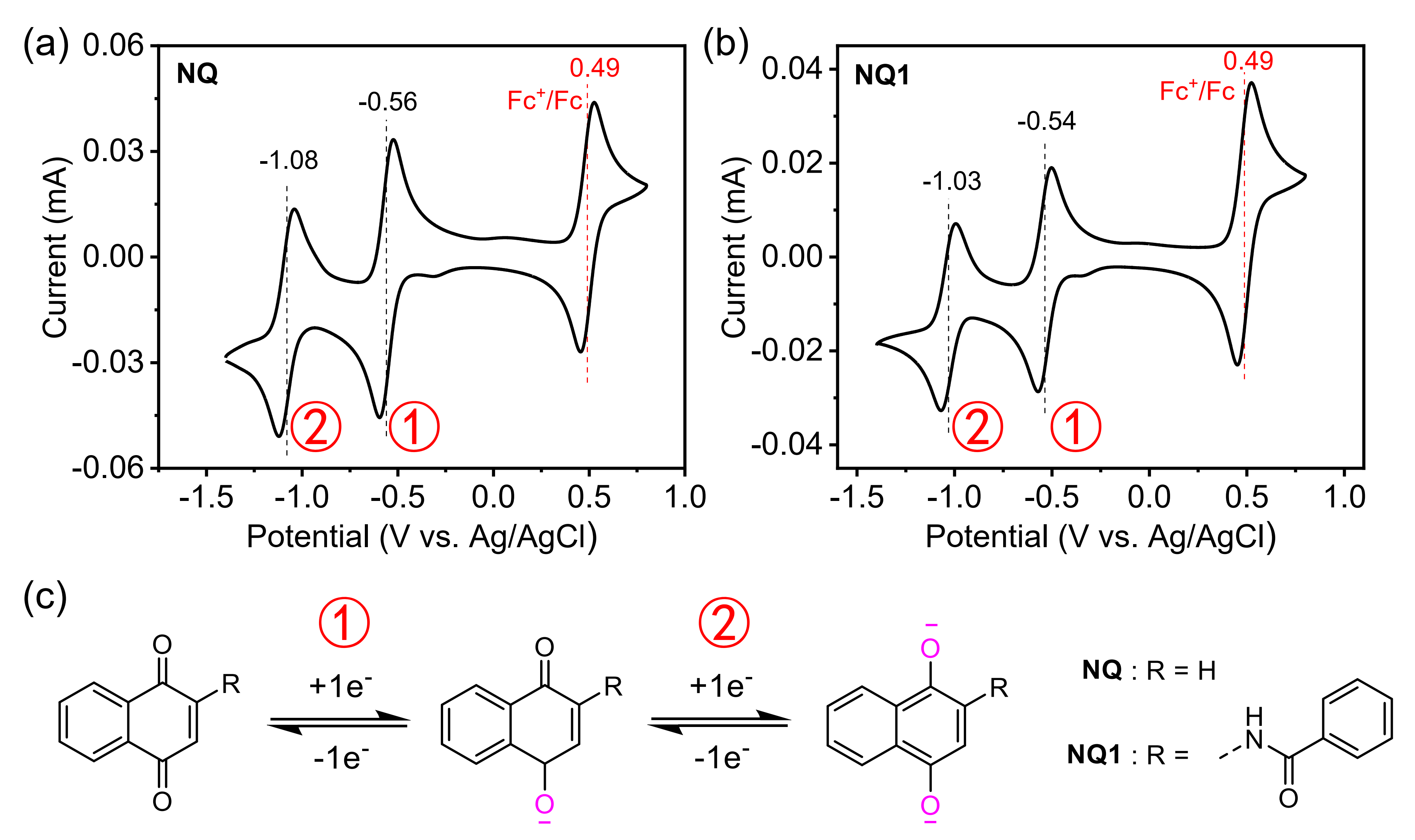


**Figure S11**. Cyclic voltammogram (CV) of (a) NQ and (b) NQ1 in acetonitrile solution (c = 1 mM) with 0.1 M TBAPF_6_ as the electrolyte at 100 mV s^-1^. Fc/Fc^+^ was used as internal reference. (c) Two redox processes for NQ and NQ1.


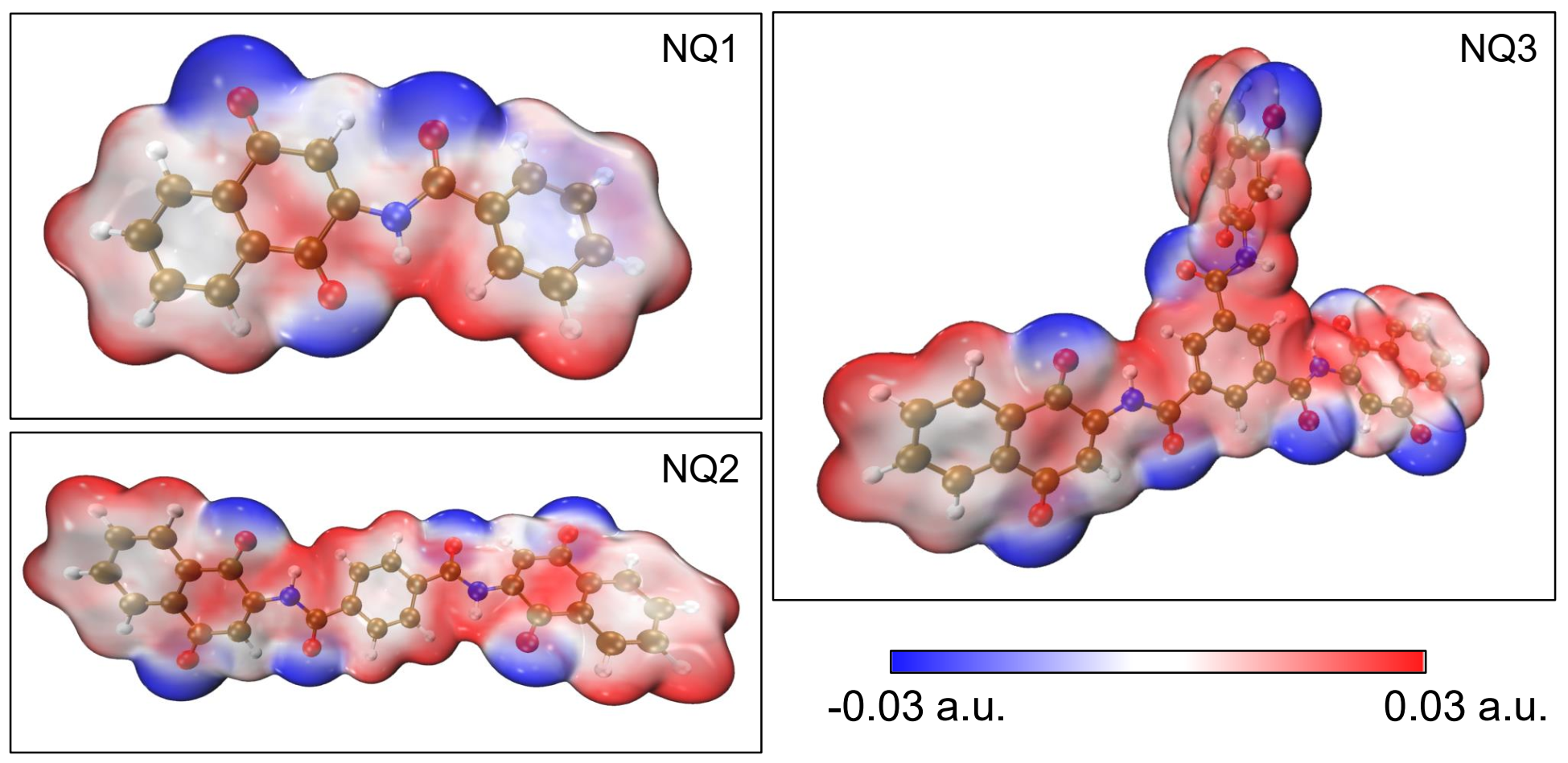


**Figure S12**. Electrostatic potential (ESP) mappings of NQ1-NQ3.


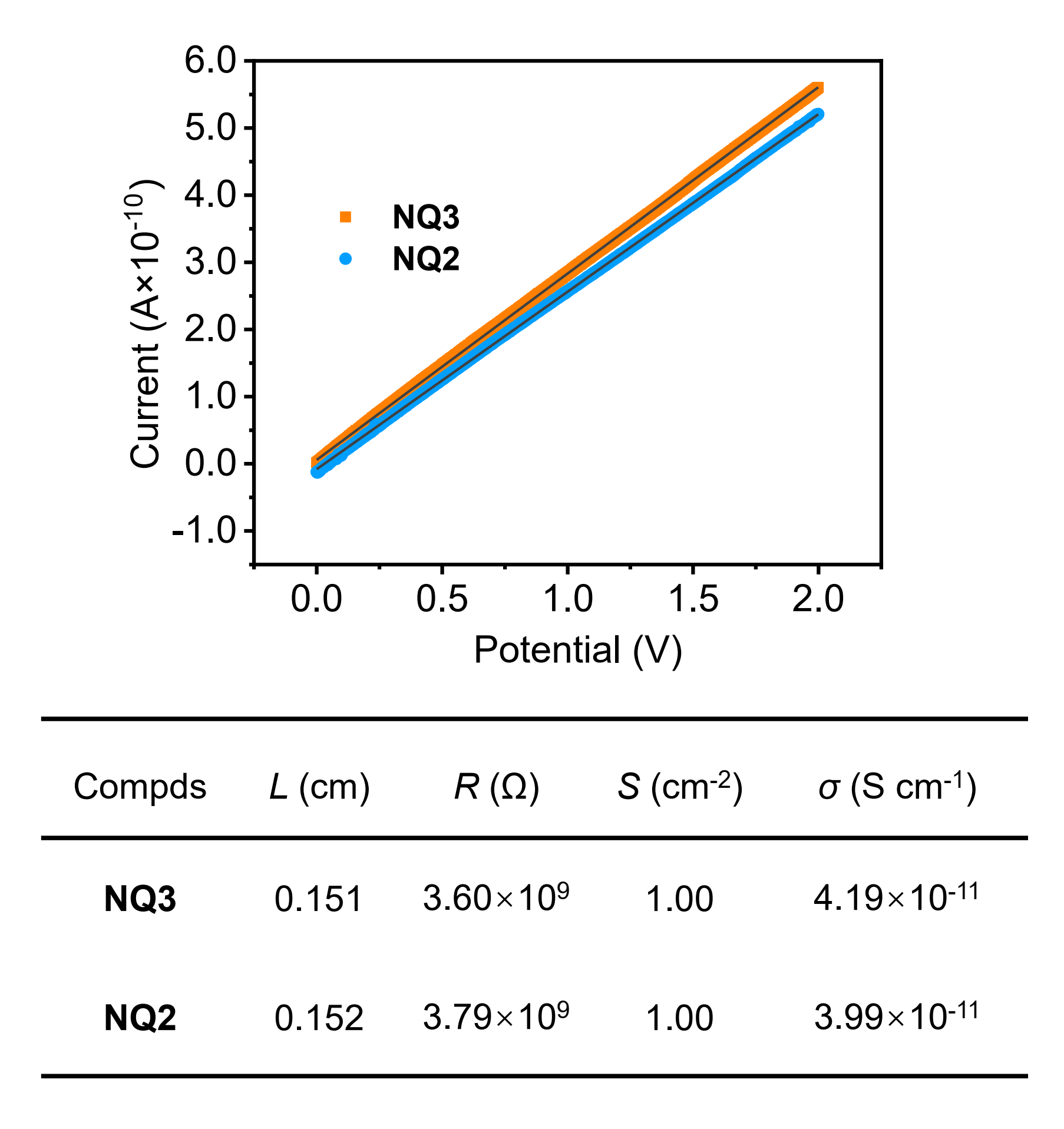


**Figure S13**. (Top) *I–V* plot of NQ2 and NQ3 pellets for measuring electrical conductivity, (bottom) key parameters for determining the electrical conductivity of NQ2 and NQ3. The electrical conductivity (σ) is determined according to the equation of σ = *L*/(*RS*), where *R*, *S* and *L* represent the resistance, surface area and thickness of the pellet.


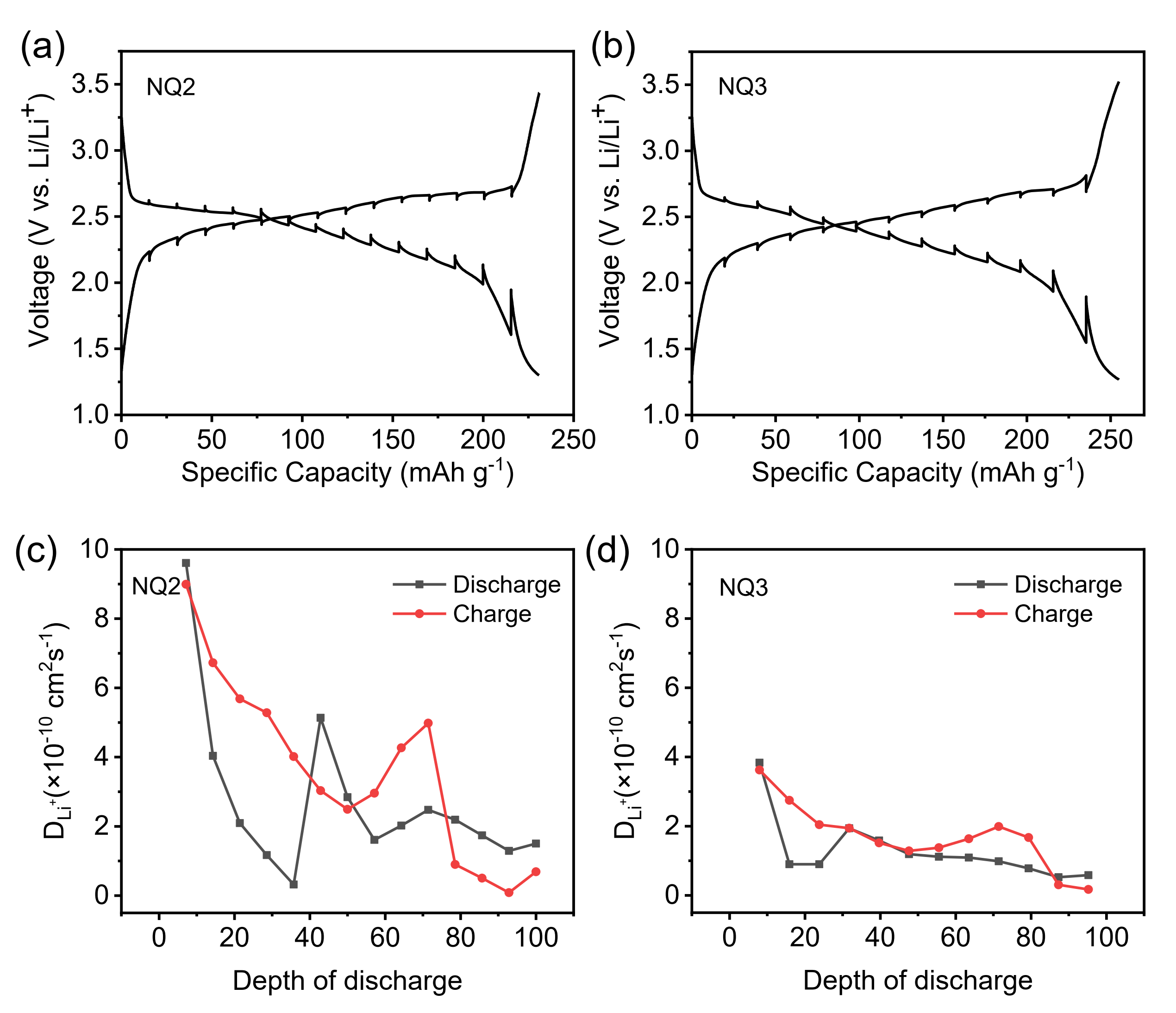


**Figure S14**. GITT curves at 0.1 A g^-1^ for (a) NQ2 and (b) NQ3.


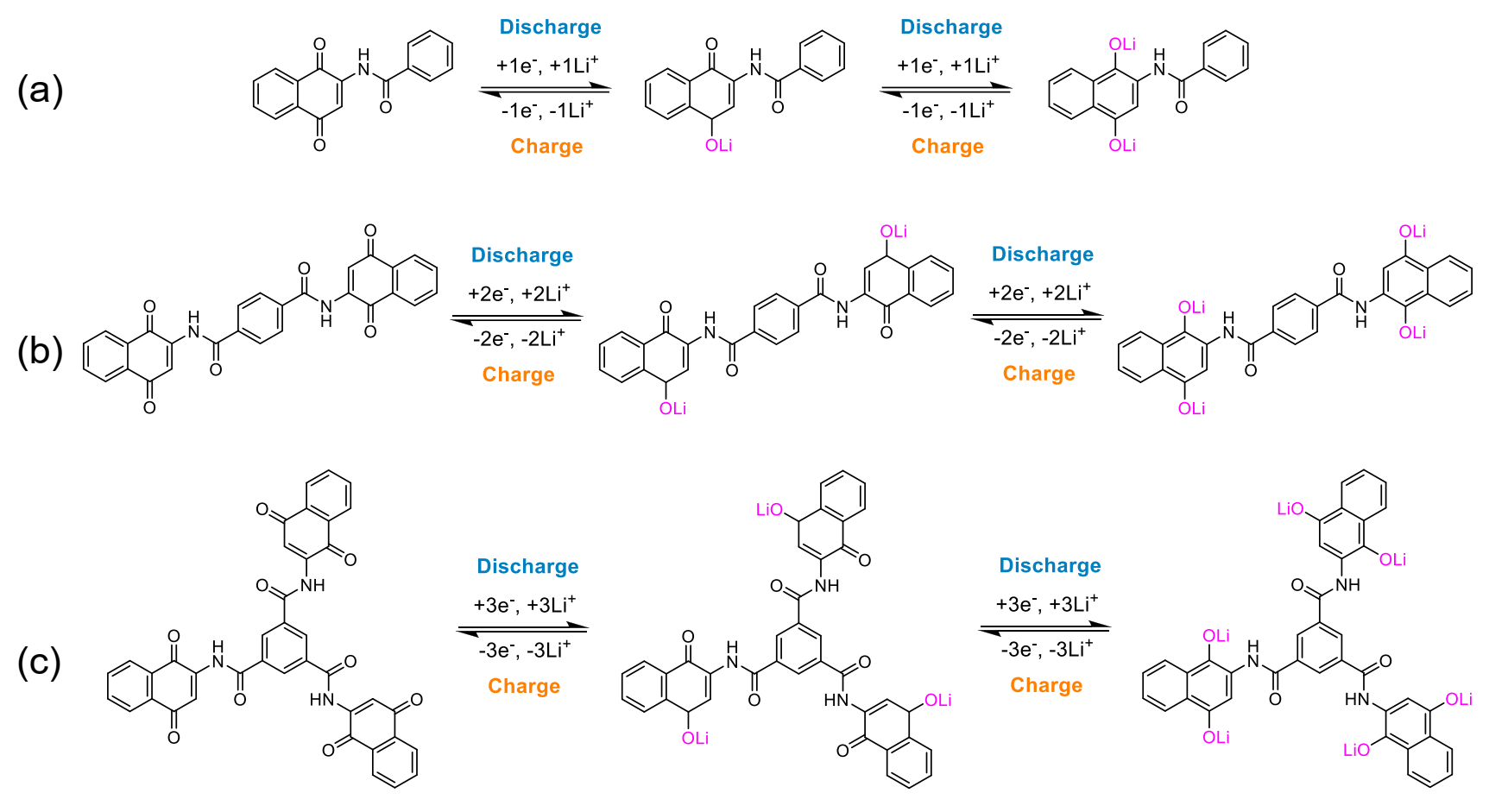


**Figure S15**. The electrochemical redox reactions of (a) NQ1, (b) NQ2 and (c) NQ3.


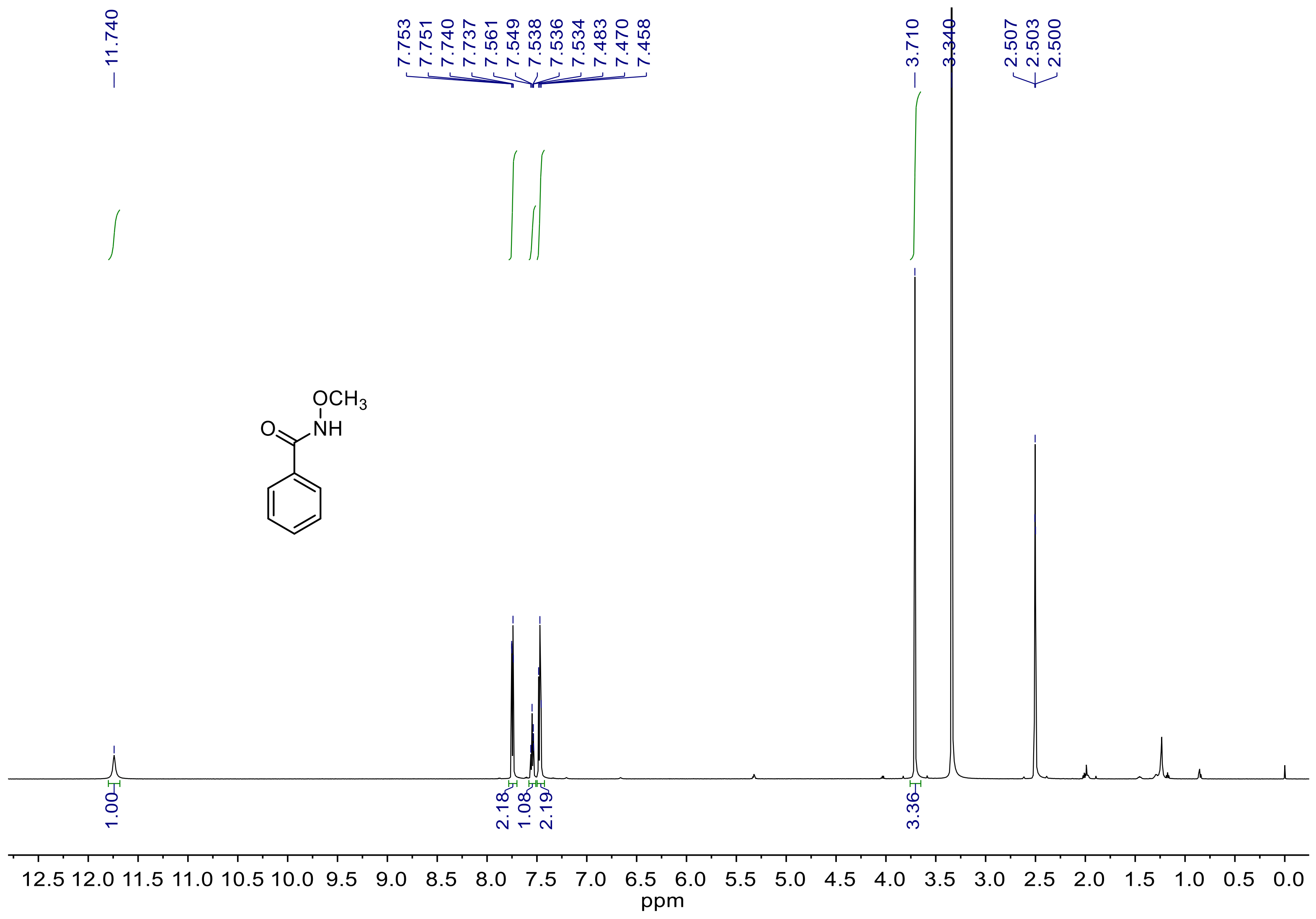


**Figure S16**. ^1^H NMR spectrum of S1 in *d*6-DMSO.


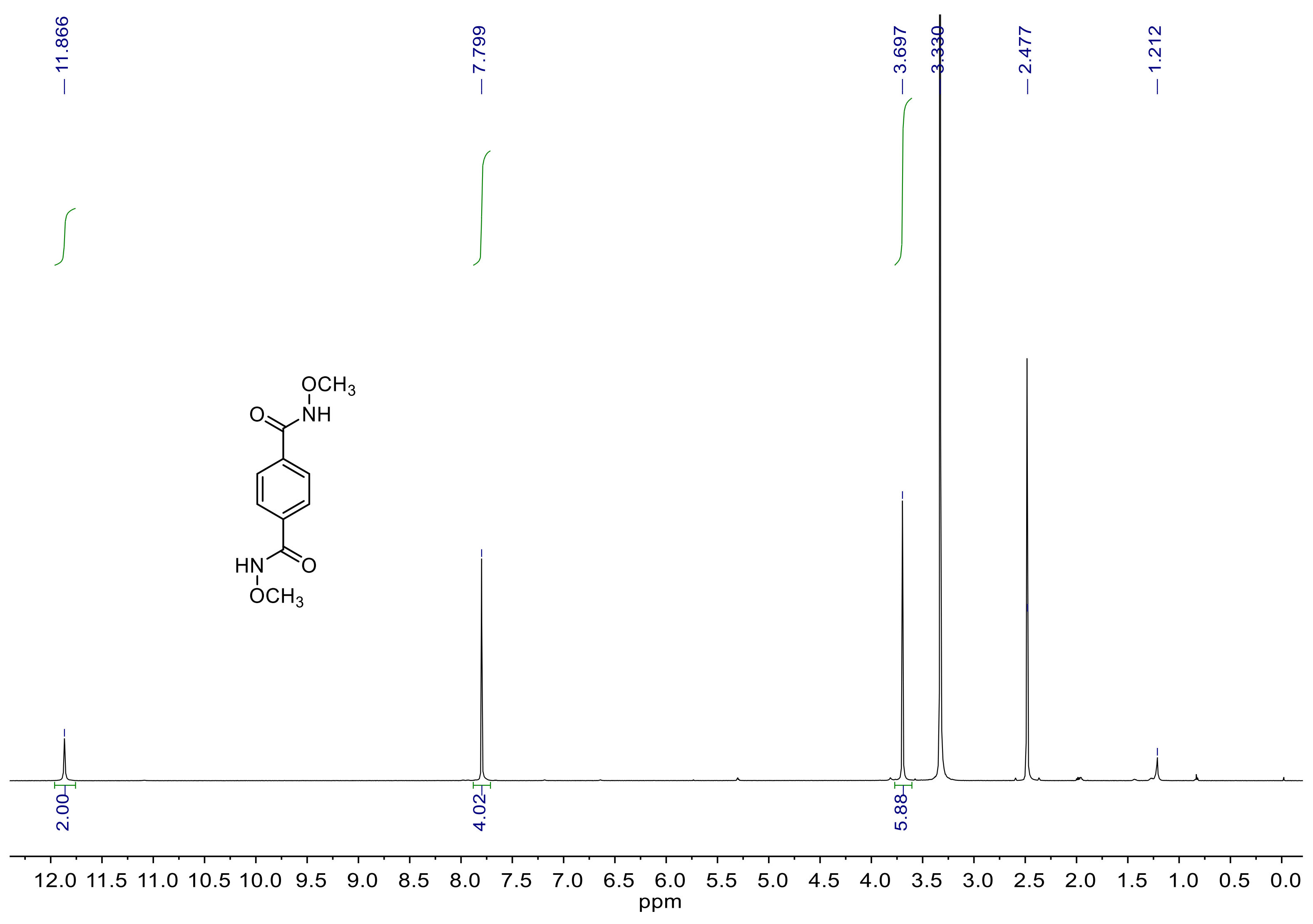


**Figure S17**. ^1^H NMR spectrum of S2 in *d*6-DMSO.


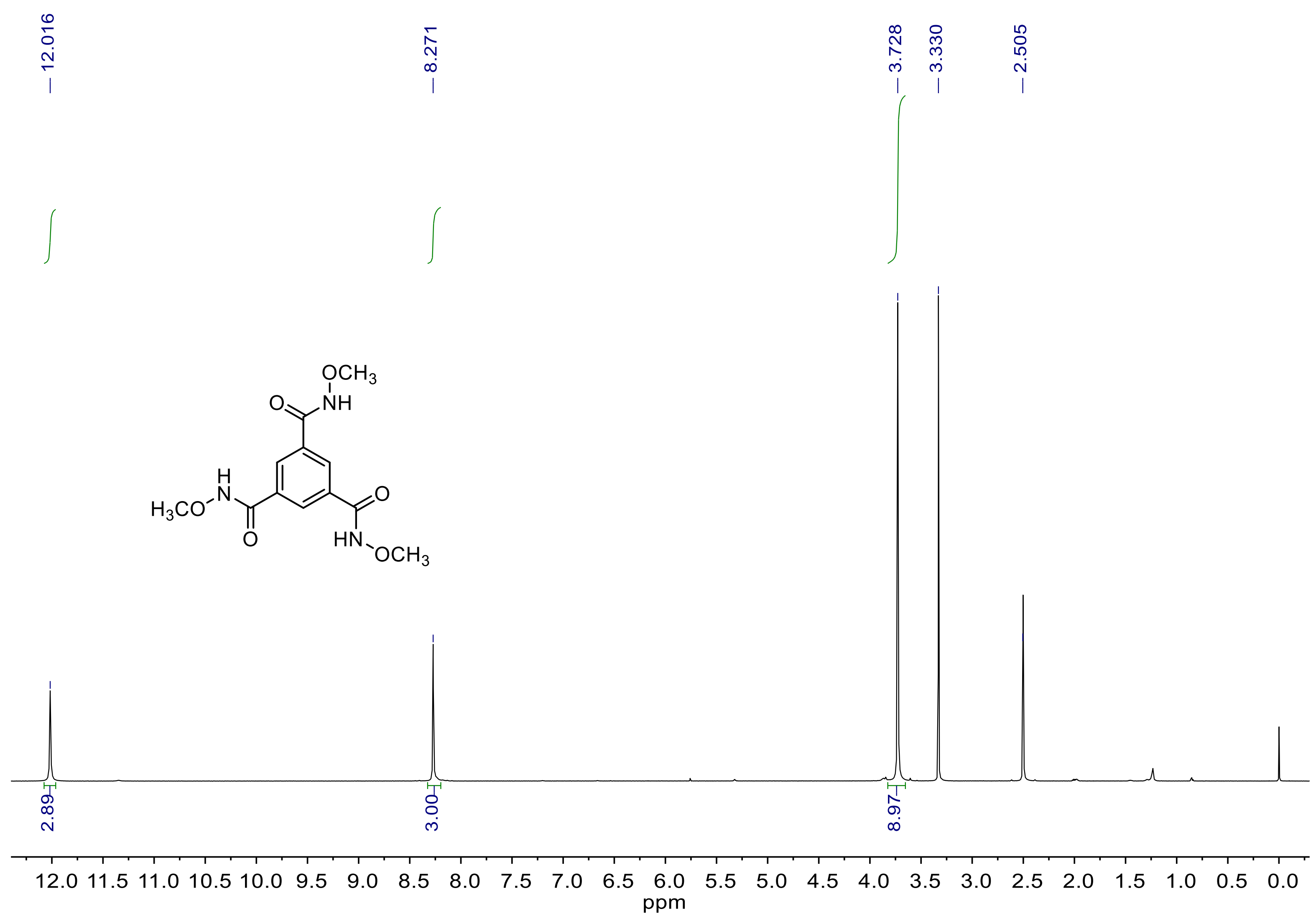


**Figure S18**. ^1^H NMR spectrum of S3 in *d*6-DMSO.

**Table S2.** Electrochemical comparison of current work and other reported quinone-based small molecular cathodes in the literature for LOBs.

| **Materials**  (reference) | **Electrode Composition** | ***C*_theor_**  (mAh g^-1^) | **Specific capacity**  (mAh g^-1^)  (**Current**) | **Cycle Stability**  Retention /Cycles / Current |
| --- | --- | --- | --- | --- |
|   **NQ1**  This work | Active materials: ketjin black: PVDF = 6: 3: 1 | 193 | 148 (0.1 A g^-1^)  119 (0.2 A g^-1^)  104 (0.4 A g^-1^)  100 (0.6 A g^-1^)  93 (1.0 A g^-1^)  87 (2.0 A g^-1^) | 106(74%)/100/0.1 A g^-1^  38(65%)/1000/2.0 A g^-1^ |
|   **NQ2**  This work | Active materials: ketjin black: PVDF = 6: 3: 1 | 225 | 172 (0.1 A g^-1^)  143 (0.2 A g^-1^)  121 (0.4 A g^-1^)  115 (0.6 A g^-1^)  107 (1.0 A g^-1^)  96 (2.0 A g^-1^) | 154(89%)/100/0.1 A g^-1^  83(46%)/1000/2.0 A g^-1^ |
|   **NQ3**  This work | Active materials: ketjin black: PVDF = 6: 3: 1 | 238 | 224 (0.1 A g^-1^)  208 (0.2 A g^-1^)  192 (0.4 A g^-1^)  188 (0.6 A g^-1^)  176 (1.0 A g^-1^)  162 (2.0 A g^-1^) | 212(95%)/100/0.1 A g^-1^  127(82%)/1000/2.0 A g^-1^ |
|   *J. Mater. Chem. A* **2018**, *6*, 3134-3140. | Active materials: Super P: PVDF = 6: 3: 1 | 467 | 387 (0.1 C)  337 (0.2 C)  297 (0.5 C)  263 (1 C)  1C=467 mAh g^-1^ | 213(55%)/20/0.1 C  37(11%)/100/1 C |
|   *Angew. Chem. Int. Ed.* **2013**, *52*, 9162-9166. | Active material: porous carbon black spheres: SWCNTs: PVDF = 62:30:3:5 | 446 | 420 (0.2 C)  380 (0.3 C)  320 (0.5 C)  220 (1 C)  1C=467 mAh g^-1^ | 379(89.8%)/100/0.2 C |
|   **BBQ**  *Adv. Funct. Mater.* **2020**, 1909597. | Active materials: GE: PVDF = 6: 3: 1 | 501 | 292 (0.1 C) | 149 (51%)/100/0.1C |
|   **BBQB**  *Adv. Funct. Mater.* **2020**, 1909597. | Active materials: GE: PVDF = 6: 3: 1 | 370 | 367 (0.1 C)  320 (0.3 C)  305 (0.5 C)  290 (0.7 C)  270 (1 C)  208 (2 C)  171 (2 C) | 306 (83%)/100/0.1C |
|   **TBQB**  *Adv. Funct. Mater.* **2020**, 1909597. | Active materials: GE: PVDF = 6: 3: 1 | 406 | 395 (0.1 C) | 217(55%)/100/1C |
|   **1,2-PNQ**  *Adv. Funct. Mater.* **2022**, 2112225. | Active material is combined with a carbon paper. | 236 | ~170 (1 C) | ~145(85%)/100/1C |
|   **1,3-PNQ**  *Adv. Funct. Mater.* **2022**, 2112225. | Active material is combined with a carbon paper. | 236 | ~175 (1 C) | ~145(82%)/100/1C |
|   **1,4-PNQ**  *Adv. Funct. Mater.* **2022**, 2112225. | Active material is combined with a carbon paper. | 236 | 199 (0.1 C)  189 (0.2 C)  180 (0.5 C)  175 (1 C)  169 (2 C)  163 (5 C)  1 C = 236 mA g-1 | 185(80%)/100/1C  ~180 (93.5%)/500/5C |
|   **NQ**  *Adv. Funct. Mater.* **2022**, 2112225. | Active material is combined with a carbon paper. | 339 | ~200 (1 C) | 103(~50%)/100/1C |
|   **IMNQ**  *Chem. Mater.* **2016**, 28, 2408−2416. | Active materials: Super P: PVDF = 6: 3: 1 | 270 | 240 (1 C) | 145(60.4%)/100/1C |
|   **DABTTO**  *ACS Appl. Mater. Interfaces.* **2022**, 14, 27994−28003 | Active materials: KB: PTFE= 6:3:1 | 473 | 273 (0.01 A g^-1^) | 117(43%)/100/0.1 A g^-1^ |
|   **MNQ**  Small. **2024**, 20, 2308881 | MNQ/CNTs | 339 | 286 (0.5 C)  188 (2 C) | 100%/100/0.5C  49%/1000/1C |
|   **TKL**  *ACS Sustainable Chem. Eng.* **2019**, 7, 13836−13844 | Active materials: Conductive Carbon Black = 1:1 | 146 | 240 (0.025 A g^-1^)  150 (0.05 A g^-1^) | 100(66.7%/300/0.05 A g^-1^ |
|   **H_2_bhnq**  *Inorg. Chem. Front.* **2014**, 1, 193–199 | Active materials: Conductive Carbon Black：PVDF = 4:5:1 | 309 | 285.6 (0.1 C) | 75(26.38%)/50/0.1 C |
|   **DNP-Li: 1**  *Commun. Mater*. **2020**, 1, 70 | Active materials: Acetylene black：PTFE =4:5:1 | 531 | 403 (0.02 A g^-1^) | 39(9.7%)/100/0.02 A g^-1^ |
|   **DNP-Li: 2**  *Commun. Mater.* **2020**, 1, 70 | Active materials: Acetylene black：PTFE =4:5:1 | 462 | 416 (0.02 A g^-1^) | 299(72.1%)/100/0.02 A g^-1^ |
|   **1-Cl_2_**  *J. Mater. Sci.* **2017**, 52, 12401–12408 | Active materials: acetylene black：PTFE =4:5:1 | 396 | 349 (0.02 A g^-1^) | 198(57%)/20/0.02 A g^-1^ |
|   **1-Cl_4_**  *J. Mater. Sci.* **2017**, 52, 12401–12408 | Active materials: Acetylene black：PTFE =4:5:1 | 316 | 274 (0.02 A g^-1^) | 180(65.7%)/20/0.02 A g^-1^ |
|   **TDMNQ**  *ACS Appl. Energy Mater.* **2024**, 7, 9134−9141 | Active materials: Ketjen Black：PVDF =5:4:1 or 6:3:1 | 225 | 201 (0.1 A g^-1^)  96 (10 A g^-1^) | 90(50%)/2500/5 A g^-1^ |

**References**

[S1] Frisch, M. J.; Trucks, G. W.; Schlegel, H. B.; Scuseria, G. E.; Robb, M. A.; Cheeseman, J. R.; Scalmani, G.; Barone, V.; Petersson, G. A.; Nakatsuji, H.; Li, X.; Caricato, M.; Marenich, A. V.; Bloino, J.; Janesko, B. G.; Gomperts, R.; Mennucci, B.; Hratchian, H. P.; Ortiz, J. V.; Izmaylov, A. F.; Sonnenberg, J. L.; Williams-Young, D.; Ding, F.; Lipparini, F.; Egidi, F.; Goings, J.; Peng, B.; Petrone, A.; Henderson, T.; Ranasinghe, D.; Zakrzewski, V. G.; Gao, J.; Rega, N.; Zheng, G.; Liang, W.; Hada, M.; Ehara, M.; Toyota, K.; Fukuda, R.; Hasegawa, J.; Ishida, M.; Nakajima, T.; Honda, Y.; Kitao, O.; Nakai, H.; Vreven, T.; Throssell, K.; Montgomery, J. A., Jr.; Peralta, J. E.; Ogliaro, F.; Bearpark, M. J.; Heyd, J. J.; Brothers, E. N.; Kudin, K. N.; Staroverov, V. N.; Keith, T. A.; Kobayashi, R.; Normand, J.; Raghavachari, K.; Rendell, A. P.; Burant, J. C.; Iyengar, S. S.; Tomasi, J.; Cossi, M.; Millam, J. M.; Klene, M.; Adamo, C.; Cammi, R.; Ochterski, J. W.; Martin, R. L.; Morokuma, K.; Farkas, O.; Foresman, J. B.; Fox, D. J., Gaussian, Inc., Wallingford, CT, USA, **2016**.

[S2] Lindström, H.; Södergren, S.; Solbrand, A.; Rensmo, H.; Hjelm, J.; Hagfeldt, A.; Lindquist, S.-E. Li^+^ Ion Insertion in TiO_2_ (Anatase). 2. Voltammetry on Nanoporous Films. *J. Phys. Chem. B* **1997**, *101*, 7717-7722.

[S3] Liu, T. C.; Pell, W. G.; Conway, B. E.; Roberson, S. L. Behavior of Molybdenum Nitrides as Materials for Electrochemical Capacitors: Comparison with Ruthenium Oxide. *J. Electrochem. Soc.* **1998**, *145*, 1882-1888.

[S4] Li, H.; Chen, L.; Xing, F.; Miao, H.; Zeng, J.; Zhang, S.; He, X. Cross-linking enhances the performance of four-electron carbonylpyridinium based polymers for lithium organic batteries. *Chem. Sci.* **2024**, *15*, 14399-14405.
